# Dual-Layer PSO-Enhanced Federated Heterogeneous Data Fusion for Hemodialysis Complication Prediction

**DOI:** 10.3390/s26165209

**Published:** 2026-08-17

**Authors:** Chihhsiong Shih, Cheng-Hsu Chen, Xiuyuan Yeah

**Affiliations:** 1 Department of Computer Science, Tunghai University, Taichung 40704, Taiwan; sefgsefg@gmail.com; 2 Department of Post-Baccalaureate Medicine, College of Medicine, National Chung Hsing University, Taichung 40227, Taiwan; cschen920@nchu.edu.tw; 3 Division of Nephrology, Department of Internal Medicine, Taichung Veterans General Hospital, Taichung 40705, Taiwan; 4 College of Medicine, National Chung Hsing University, Taichung 40227, Taiwan; 5 Department of Life Science, Tunghai University, Taichung 40704, Taiwan

**Keywords:** Medical IoT, federated learning, hemodialysis, heterogeneous data fusion, Particle Swarm Optimization, edge computing

## Abstract

**Highlights:**

**What are the main findings?**

- We formulated hemodialysis monitoring as a federated sensor fusion problem in a Medical IoT architecture, where heterogeneous multimodal sensors and privacy constraints preclude centralized learning.
- We designed a dual-layer PSO-enhanced federated learning framework that optimizes CNN-based sensor feature weights at the client level and aggregation weights at the server level, explicitly addressing heterogeneity in both features and client performance.

**What are the implications of the main findings?**

- Through validation of the framework on real hemodialysis datasets with non-IID client partitions, we demonstrated substantial improvements in hemodialysis complication prediction accuracy and F1-score compared with baseline FedAvg and single-layer PSO variants, along with robust cross-validation results.
- We highlighted the practical feasibility of deploying the proposed method on edge gateways attached to hemodialysis machines in real clinical settings through an ablation analysis and resource-awareness discussion.

**Abstract:**

Taiwan has one of the highest dialysis prevalences worldwide, making safe and reliable hemodialysis monitoring a critical sensor-based healthcare challenge. Modern hemodialysis machines integrate heterogeneous multimodal sensors (pressure, flow, conductivity, temperature, and cardiovascular signals), but differences in machine brands, data formats, and privacy constraints hinder centralized learning and robust complication prediction. This work proposes a Medical IoT-oriented federated learning framework, PSOFed-HD, that performs dual-layer Particle Swarm Optimization (PSO) to enhance heterogeneous sensor fusion for predicting dialysis-related hypotension and discomfort events. The events are defined as abnormal blood-pressure states, defined as systolic blood pressure <90 mmHg. Each hemodialysis machine is paired with an edge gateway acting as an FL client, where local PSO optimizes CNN feature weights over non-IID sensor subsets, while the central server applies PSO-driven aggregation to adaptively weight client models according to validation performance. Experiments on real-world hemodialysis datasets with 17 most commonly seen HD physiological features demonstrate that standard FedAvg yields an accuracy of 65.24% and F1-score of 0.5318, server-side PSO improves accuracy to 75.11%, and client-side PSO further raises accuracy to 81.97%. The proposed dual-layer PSO framework achieves the best performance, with 90.56% accuracy and an F1-score of 0.8533, along with superior ROC characteristics (AUC = 0.908) and stable cross-validation across 11 folds. State-of-the-art federated learning techniques for non-IID data such as SCAFFOLD and FedProx are also examined using the same heterogeneous HD dataset. The performance is close to our client-only PSO techniques, proving the merits of our dual-layer PSO architecture. These results confirm that jointly optimizing local feature representations and global aggregation weights enables effective fusion of heterogeneous hemodialysis sensor data under privacy-preserving Medical IoT constraints, providing a practical decision-support approach for real-time complication prediction in dialysis units. Future work will incorporate temporal models such as LSTM or Transformer architectures to achieve early event prediction.

## 1. Introduction

The global rise in chronic kidney disease (CKD) and end-stage renal disease (ESRD) has made maintenance hemodialysis an indispensable life-sustaining therapy, particularly in countries such as Taiwan where dialysis prevalence ranks among the highest worldwide. During hemodialysis, patients frequently experience complications including intradialytic hypotension, nausea, muscle cramps, and dizziness, which can degrade quality of life, interrupt treatment, and trigger emergency interventions. Real-time complication detection and risk monitoring of these adverse events would allow clinicians and nursing staff to adjust treatment parameters in real time, improving safety and reducing the clinical burden in dialysis units.

Modern hemodialysis machines operate as complex Medical IoT devices, integrating heterogeneous multimodal sensors that continuously monitor physiological and process variables such as arterial and venous pressure, blood and dialysate flow, conductivity, temperature, air bubbles, and related chemical indicators. These sensor streams form rich time-series data describing patient status and machine operation, and they are crucial for detecting abnormalities and preventing complications. However, differences in machine brands, hardware configurations, and data formats across machines lead to heterogeneous feature sets and non-uniform distributions, which complicate centralized data collection and model training. At the same time, strict requirements for patient privacy and regulatory compliance discourage the sharing of raw medical data, limiting the feasibility of conventional cloud-based analytics.

Federated learning (FL) has emerged as a promising paradigm for privacy-preserving model training in distributed Medical IoT environments. In FL, each client performs local training on its own data and uploads only model parameters to a central server for aggregation, thus avoiding direct transmission of raw patient records. This is well suited to networks of hemodialysis machines and edge gateways, where each client sees only a subset of sensor modalities and local patient cohorts. Nevertheless, standard aggregation schemes such as Federated Averaging (FedAvg) treat client models as homogeneous and typically weight them by sample size, which can result in biased global models and degraded performance when data and feature distributions are highly non-IID. In particular, naive weight averaging does not explicitly address heterogeneous sensor fusion, and may underutilize informative clients or overemphasize biased ones.

To address these challenges, this study focuses on real-time prediction of dialysis-related complications using a federated framework that explicitly targets heterogeneous sensor fusion in hemodialysis Medical IoT systems. We collect real-world hemodialysis records comprising 17 features from multiple sensors and treatment parameters, labeled according to blood pressure behavior (normal vs. abnormal). The Normal (0) labels indicate that the patient’s blood pressure fluctuations remain within the normal range. Abnormal (1) indicate that the patient experiences abnormal blood pressure drops, falling below 90 mmHg (systolic blood pressure) as measured bedside. The data are partitioned into multiple clients that each observe only partial feature subsets, thereby simulating realistic differences between machine configurations and sensor availability. On top of a 1D convolutional neural network (CNN) for time-series feature extraction, we introduce a dual-layer Particle Swarm Optimization (PSO) mechanism: client-side PSO for feature-level weighting of intermediate sensor representations, and server-side PSO for dynamic optimization of aggregation weights across heterogeneous client models. By jointly enhancing local feature discrimination and global aggregation, the proposed framework aims to improve real-time prediction accuracy and stability under non-IID conditions while preserving data privacy.

Most current FL methods rely on aggregation algorithms such as FedAvg to combine client models, but these approaches perform poorly under data heterogeneity. To address this issue, Particle Swarm Optimization (PSO)—a swarm intelligence-based optimization algorithm—has been introduced to adjust aggregation weights. PSO simulates the cooperative search behavior of particles, enabling more effective fusion of client model parameters and improving classification accuracy. This research proposes a two-stage PSO adaption on model parameters to enhance the final prediction accuracy on the hemodialysis treatment discomfort, one on the client side, the other on the server side.

When PSO was applied at both the client and server levels, the initial accuracy (around the third epoch) already exceeded that of the other two methods, starting at approximately 50%.

As training progressed, the accuracy curve exhibited a steeper and more stable upward trend, surpassing 85% around the 10th epoch and stabilizing near 90% by the 20th epoch. Overall, the dual-layer PSO method of PSOFed-HD achieved the highest final accuracy, outperforming the baseline and server-only PSO methods significantly.

State-of-the-art federated learning techniques for non-IID data such as SCAFFOLD and FedProx are also examined using the same heterogeneous HD dataset. A variety of evaluations are performed on these techniques with our proposed PSO-based methods including client accuracy, accuracy per training cycles, ROC etc. Statistical analyses with respect to our PSO-based optimization methods are performed. Results show a significance level over these recently proposed non-IID handling federated learning techniques.

These findings indicate that the dual-layer PSO approach can more effectively integrate heterogeneous client features and dynamically adjust aggregation weights, ultimately achieving superior classification performance in federated learning environments with non-IID data distributions.

While current CNN-based dual-layer PSO architecture emphasizes real-time detection of HD complication events on edge devices attached to the hemodialysis machine, future work will incorporate temporal models such as LSTM or Transformer architectures to achieve true early prediction.

## 2. Background and Related Work

### 2.1. Hemodialysis Treatment Principles and the Role of Hemodialysis Machine Sensors

Hemodialysis treatment goals include toxin clearance such as urea nitrogen (BUN), creatinine, etc. Fluid balance controls dry weight and prevents pulmonary edema. Electrolyte stability maintains blood potassium, sodium, and acid-base balance. Symptom improvement reduces complications like hypotension, cramps, and nausea during dialysis. The treatment principles are introduced below:

Diffusion: The components of the dialysate are carefully formulated, with concentrations lower than those of toxins like urea and creatinine in the blood. This creates a concentration gradient across the semipermeable membrane of the dialyzer, allowing small-molecule toxins to diffuse from the blood into the dialysate.

Ultrafiltration: The dialysis machine uses transmembrane pressure (TMP) to push excess water out of the blood and into the dialysate side. This is key for controlling the patient’s dry weight and preventing edema and high blood pressure.

Convection: During water movement, some middle-molecule toxins (like β2-microglobulin) are carried away with the fluid. Convection complements diffusion, enhancing the clearance efficiency.

### 2.2. Clinical Functional Modules of the Dialysis Machine

A regular dialysis machine consists of the following major components:

Blood pump: Maintains the blood flow rate (usually 200–400 mL/min) to ensure stable circulation.

Dialysate delivery system: Controls dialysate flow rate and temperature, and monitors electrolyte concentrations.

Pressure sensors: Monitor arterial pressure, venous pressure, and transmembrane pressure to prevent line blockage or membrane rupture.

Air/bubble detector: Prevents air from entering the bloodstream to avoid air embolism.

Conductivity and pH detection: Ensures dialysate composition is correct to prevent metabolic acidosis or alkalosis.

Temperature control: Keeps the dialysate at 36–37 °C to avoid hypothermia or hyperthermia. Blood pressure and heart rate monitoring: Reflect the patient’s tolerance in real time. Hemodialysis machines rely on a suite of integrated sensors to ensure safe, precise, and effective treatment. The most critical sensors include pressure, temperature, flow, force, and air/bubble detectors, each serving a distinct role in monitoring blood and dialysate conditions.

### 2.3. Basic Federated Learning Method: FedAvg

#### 2.3.1. Model Structure

All clients use the same convolutional neural network (CNN) architecture, consisting of a 1D convolutional layer, a flatten layer, a fully connected layer, and a dropout layer, followed by a sigmoid activation function with a 0.5 threshold for binary classification.

#### 2.3.2. Training Objective

As shown in Equation (1), model weights from all clients are aggregated using the Federated Averaging (FedAvg) algorithm to form the global model, which serves as the control group baseline for comparison.

(1) $$w^{\mathrm{global}}=\sum_{k=1}^{K}\frac{n_{k}}{N}w_{k}$$

$w_{k}$: Model weights of the *k*-th client.
$n_{k}$: Number of samples on the *k*-th client.
*N*: Total number of samples across all clients.
*K*: Total number of clients.

### 2.4. Particle Swarm Algorithm

Particle Swarm Optimization (PSO) is a population-based metaheuristic algorithm inspired by the social behavior of birds flocking or fish schooling. It is widely used for solving optimization problems. Each particle represents a candidate solution in the search space. Particles move through the space by updating their velocity and position based on:

Their own best-known position (personal best, pBest).
The best-known position among all particles (global best, gBest).

The algorithm iteratively updates particles until a stopping condition (e.g., max iterations or convergence) is met. PSO is valued for its simplicity, efficiency, and ability to handle nonlinear, multidimensional optimization problems.

### 2.5. Federated Learning (FL) for Hemodialysis Related Complication Prediction

Federated Learning (FL), first proposed by Google, was designed to enable distributed model training while preserving data privacy. This technology allows each client to train models locally and upload only the trained model parameters to a central server for aggregation, rather than transferring raw data directly. Such an approach is particularly suitable for privacy-sensitive or data-restricted applications, including smartphone behavior analysis, medical diagnosis modeling, and industrial fault prediction.

Technically, the core workflow of federated learning consists of three stages: local model training, parameter uploading and aggregation, and global model updating. The Federated Averaging (FedAvg) algorithm, introduced by Konečný et al. [1] remains the most widely adopted aggregation method, demonstrating good performance in communication efficiency and computational stability. In many practical implementations, FL also supports asynchronous updates, partial client participation, and mini-batch training mechanisms, further reducing training time and communication overhead.

Despite its advantages in distributed training and privacy preservation, FL still faces challenges during deployment due to the data heterogeneity of clients, especially in the hemodialysis environment. These practical issues form the foundation for ongoing research and are a key motivation behind the framework design in this study.

Some relevant federated learning techniques have been used in the hemodialysis treatment-related complications from minor discomfort to major lethal events. Chun-Te Huang et al. [2] federated learning (FL), to establish an aggregate model for acute kidney injury (AKI) prediction in critically ill patients in Taiwan. This work used data from the Critical Care Database of Taichung Veterans General Hospital (TCVGH) from 2015 to 2020 and electrical medical records of the intensive care units (ICUs) between 2018 and 2020 of four referral centers in different areas across Taiwan. AKI prediction models were trained and validated thereupon. An FL-based prediction model across hospitals was then established. The study included 16,732 ICU admissions from the TCVGH and 38,424 ICU admissions from the other four hospitals. The complete model with 60 features and the parsimonious model with 21 features demonstrated comparable accuracies using extreme gradient boosting, neural network (NN), and random forest, with an area under the receiver-operating characteristic (AUROC) curve of approximately 0.90. The Shapley Additive Explanations plot demonstrated that the selected features were the key clinical components of AKI for critically ill patients. The AUROC curve of the established parsimonious model for external validation at the four hospitals ranged from 0.760 to 0.865. NN-based FL slightly improved the model performance at the four centers.

### 2.6. Challenges of Heterogeneous Data in Federated Learning Feature Fusion

While federated learning effectively mitigates the privacy risks associated with centralized learning, it faces major challenges when dealing with heterogeneous data [3]. Heterogeneity manifests at three levels: data heterogeneity, system heterogeneity, and statistical skew. Data heterogeneity refers to differences in data sources, formats, and feature distributions among clients.

Statistical heterogeneity means uneven class distributions across clients (e.g., certain clients may have samples concentrated in a few categories).

System heterogeneity arises from variations in hardware performance, storage capacity, and battery life, which can impact synchronization and fairness during training [4]. When client data distributions differ, even using FedAvg may result in a global model that generalizes poorly or biases toward clients with more abundant data, causing unstable training or convergence issues. Niknam et al. [5] demonstrated that in wireless device scenarios, highly heterogeneous environments significantly degrade convergence and classification performance if client participation ratios and model contributions are not properly balanced.

To address these challenges, various strategies have been proposed. Some studies introduce adaptive aggregation methods that adjust client weights based on model loss or gradient magnitude [6,7], while others vary local training epochs or learning rates according to data characteristics [8,9]. In addition, edge intelligence and deep reinforcement learning techniques have been integrated into resource-constrained systems to optimize client selection and computation [9,10,11]. Furthermore, clustered FL and personalized FL approaches have been developed to handle data diversity by grouping clients or training multiple models, enhancing both local adaptability and global generalization [12,13,14].

The heterogeneity problem not only affects model accuracy but also impacts fairness and system efficiency. Therefore, achieving effective learning across non-uniform data and system conditions—while preserving privacy—has become a crucial bottleneck for FL scalability. This study aims to address this challenge through the integration of dual-layer PSO optimization, seeking to balance model performance and features fusion issues.

## 3. Materials and Methods

### 3.1. System Architecture and Medical IoT Topology

**Figure 1:**
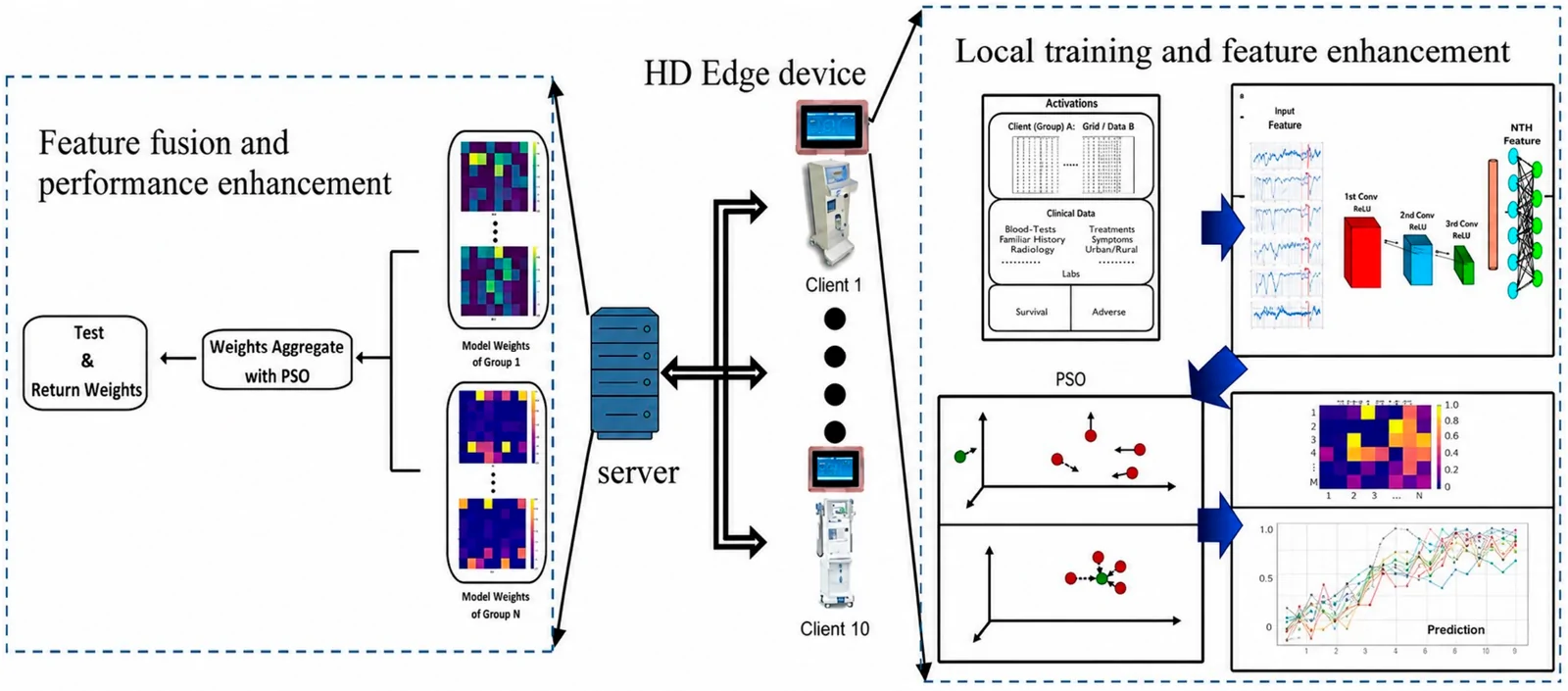
Overall system architecture (The architecture depicts a federated learning framework divided into local edge processing and centralized server aggregation. On the right, HD Edge devices, Clients 1 through 10, execute Local training and feature enhancement, where raw inputs—such as signal activations and clinical data—are processed through a multi-layer convolutional neural network as shown in the top blue arrow to extract deep features. Particle Swarm Optimization (PSO) is applied locally to fine-tune features a as pointed by the next blue arrow sign and generate local model weight updates alongside initial predictions afterwards. These updated local model weights are then sent to the central server. On the left, in the Feature fusion and performance enhancement stage, the server collects the model weight matrices across all client groups, Group 1 to Group N, and performs global Weights Aggregation with PSO. Finally, the optimized aggregated weights are tested and returned back to the edge clients to complete the collaborative training loop).

The proposed PSOFed-HD framework, as shown in Figure 1, consists of five stages: data preprocessing, client-side local training, server-side model aggregation, iterative federated updating, and final evaluation. The overall architecture is designed for a Medical IoT environment in which each hemodialysis machine or edge gateway functions as a federated client. Raw patient data remain local to each site, and only model parameters are exchanged with the central server.

During preprocessing, the original hemodialysis records are merged, labeled, normalized, and partitioned into multiple client subsets to simulate heterogeneous deployment conditions. Each client receives only a subset of the available features to reflect realistic differences in sensor availability across devices. The local models are then trained independently, and their updated parameters are sent to the server for aggregation. The server evaluates candidate global models and redistributes the best-performing one to the clients for the next training round. This cycle continues until convergence or a predefined stopping criterion is reached.

### 3.2. Hemodialysis Dataset Preprocessing

**Figure 2:**
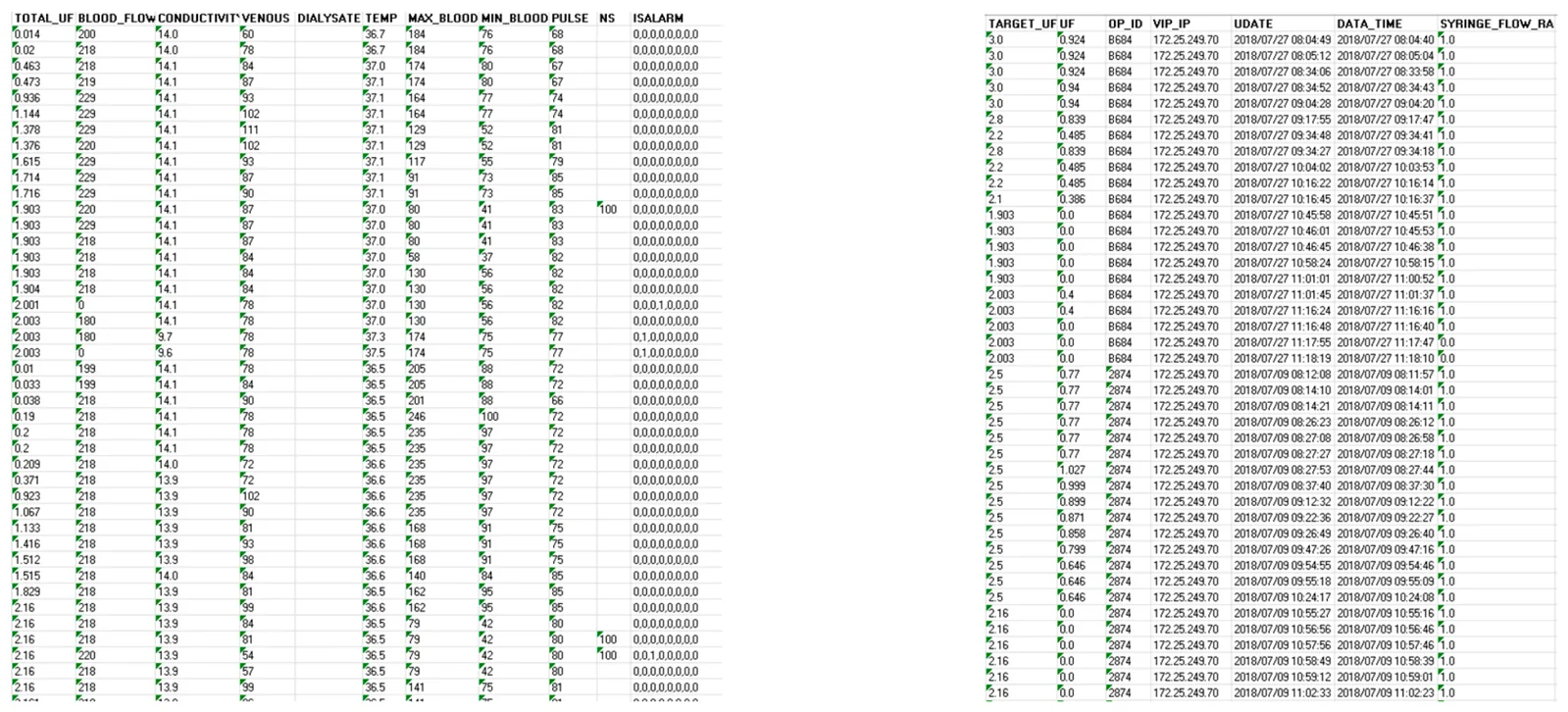
Hemodialysis data set.

The dataset used in this study was collected from real hemodialysis patient records (as shown in Figure 2). Each subfigure contains about 9 features representing various physiological indicators and measurement data recorded during the dialysis process. The dataset includes two classification labels: Normal (0): Indicates that the patient’s blood pressure fluctuations remain within the normal range; Abnormal (1): Indicates that the patient experiences abnormal blood pressure drops, falling below 90 mmHg (systolic blood pressure) as measured bedside. Each class of data set is collected from a total of ten treatment sessions on ten dialysis machines. Around 200 records are collected each session for each client machine. The data distribution is about equal in data amount between classes. The population age is around 60~70, while man/woman distribution is about even for both classes. A total of twenty two sessions of data are collected for both normal and abnormal treatments. The systolic blood pressure feature is excluded from the training and testing runs among clients, normal and abnormal alike. These data are collected during the time period of July to September 2020. An IRB permission document has been issued by Institutional Review Board I & II of Taichung Veterans General Hospital with the sequence number of SE20056A. Standard de-identified benchmark records have been adapted on both server and clients. Table 1 summarizes the statistics of the data collection process such as the number of unique sessions, records per class, participating machines or clients, feature subsets available to each client, and the numbers of observations assigned to local training, local validation, server-side validation, and final evaluation. We used treatment session as base units for records collection. The number of unique patients is not disclosed for privacy conservation purposes.

The inclusion criteria for the IRB is listed below:

- Adult patients (≥18 years old) receiving maintenance hemodialysis.
- Stable dialysis schedule (e.g., thrice weekly for ≥3 months).
- Electronic health record (EHR) availability with sufficient longitudinal data (labs, vitals, treatment parameters).
- Consent to participate in data-sharing under federated learning protocols.
- Clinical stability (no hospitalization within the past 30 days).
- Dialysis adequacy documented (e.g., Kt/V ≥ 1.2).

The Exclusion Criteria are as follows:

- Pediatric patients (<18 years old).
- Patients on peritoneal dialysis or mixed modalities.
- Recent acute kidney injury (AKI) requiring temporary dialysis.
- Unstable clinical condition (e.g., active infection, hospitalization, hemodynamic instability).
- Incomplete or poor-quality records (missing key labs, treatment logs).
- Patients with <3 months of dialysis history (insufficient longitudinal data).
- Withdrawal of consent or refusal to share data under federated learning framework.

With regard to missing data handling, we used simple statistical imputation mean/median substitution for continuous variables (e.g., hemoglobin, Kt/V) and mode substitution for categorical variables (e.g., vascular access type).

The features include those from group A and B as listed above in Table 1. Two types of hemodialysis machine made use of a subset of around nine features, mutually exclusive, during the federated training to exemplify heterogeneous fusion process.

All data were read from two separate files—one labeled as “normal” and the other as “abnormal.” The datasets were then merged and randomly shuffled to ensure an even and unbiased distribution of samples across both classes.

### 3.3. Client-Side Model

**Figure 3:**
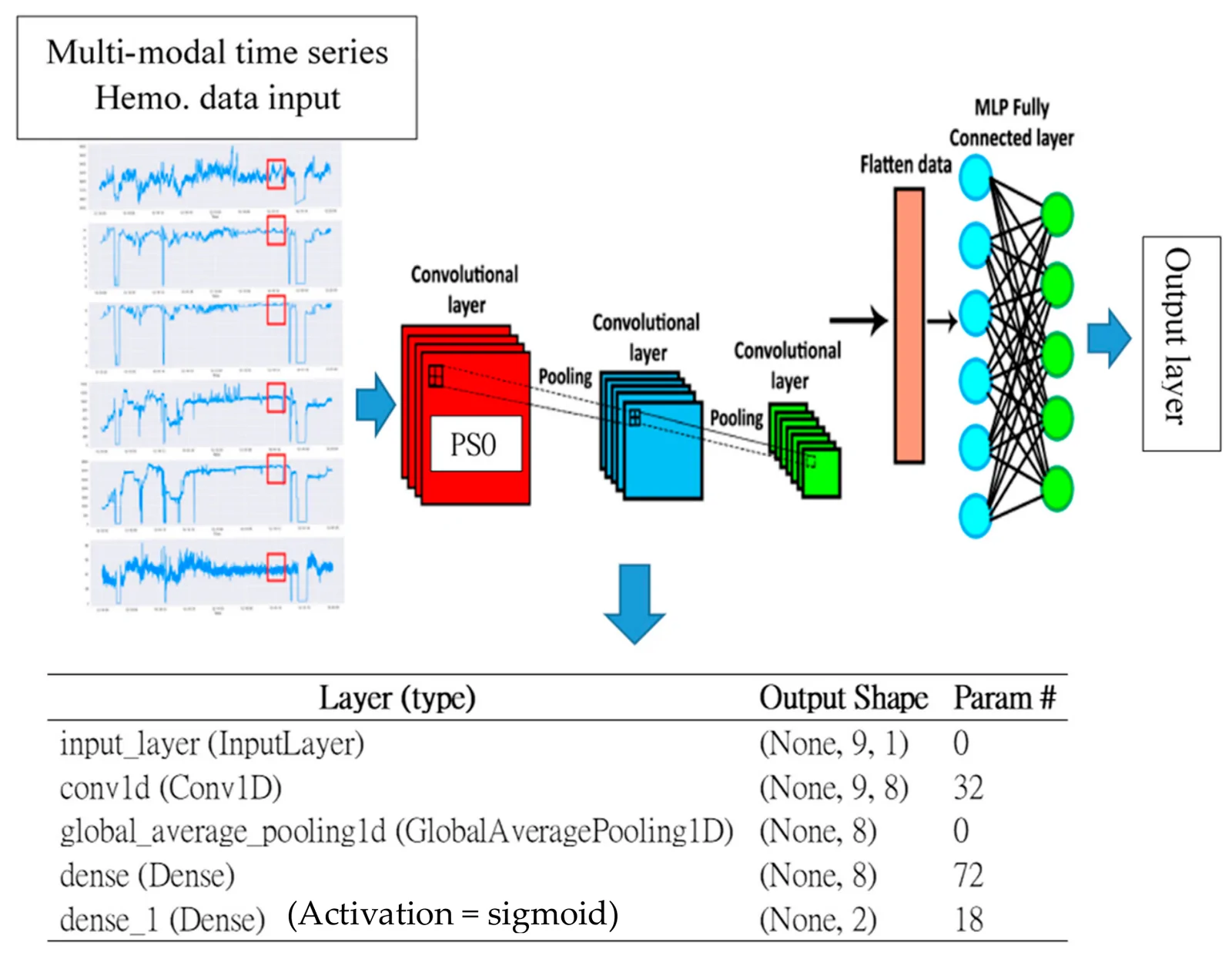
Model architecture (The left side of the figure shows HD sensor measurements data serving as input layer to the CNN model. The model then went through the model pipeline as shown in the middle arrow signs. The bottom listing is the parameters specification of each layer in the pipeline).

Figure 3 shows a 1D convolutional neural network for multi-modal hemodynamic time-series classification for each client. The input is a set of synchronized physiological signals, which are passed through convolution and pooling blocks to extract local temporal patterns, then flattened and sent to fully connected layers for final prediction.

The CNN model pipeline is as follows:

1. Multi-modal time-series input layer: multiple hemodynamic signals are used as input with a shape of (9 × 1).
2. Conv1D layer: learns temporal features such as short waveform patterns, transitions, or local anomalies with a shape of (9 × 8).
3. Flatten/global pooling: converts the learned feature maps into a vector with a shape dimension of eight.
4. Dense layers: combine the extracted features for classification with a shape dimension of eight.
5. Output layer: produces the final class prediction with a shape of two. The activation function is sigmoid with a threshold value of 0.5 for binary classification.

On each client, PSO is applied to optimize the weights of intermediate feature representations produced by the CNN. Each particle corresponds to a candidate feature-weighting vector. The fitness of each particle is evaluated on the local validation set according to classification accuracy. The best particle is retained as the client’s optimal feature fusion configuration. This mechanism enables the model to emphasize informative local features even when some modalities are missing or incomplete.

### 3.4. Data Flow for the PSO-Enhanced Federated Learning

**Figure 4:**
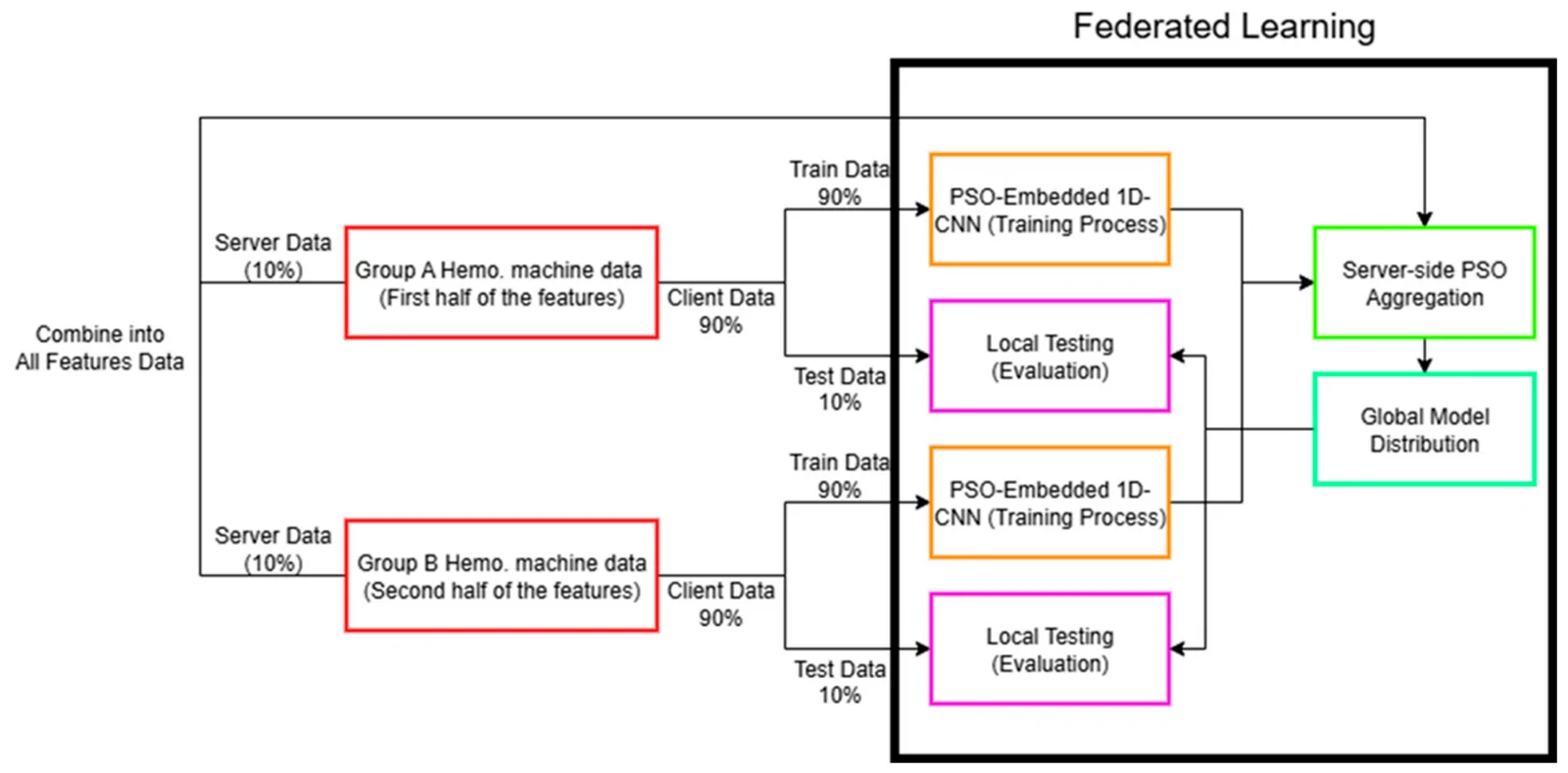
Training and testing data flow of PSOFed-HD.

Figure 4 illustrates the complete data flow of the proposed PSO-enhanced federated learning framework, covering both training and testing phases. The workflow begins at the client side, where each client receives its own local hemodialysis dataset containing heterogeneous feature subsets. Due to differences in sensor availability and dialysis machine configurations, each client only processes partial features, which reflects a realistic non-IID clinical scenario.

During local training, the raw time-series data are first normalized and fed into the 1D CNN model for initial feature extraction. At this stage, client-side PSO is applied to the intermediate convolutional feature maps, where particles represent different feature weighting combinations. Through iterative optimization, each client identifies an optimal feature weighting scheme that maximizes its local validation accuracy, thereby enhancing discriminative feature representation under limited feature conditions.

After local optimization, the updated model parameters are transmitted to the central server without sharing raw patient data, preserving data privacy. At the server side, a second PSO process is performed to optimize the aggregation weights of different client models. Each particle represents a candidate aggregation strategy, and its fitness is evaluated based on server-side validation accuracy. The best-performing particle determines the final global model, which is then redistributed to all clients for the next training round.

This dual-layer PSO data flow enables both feature-level enhancement at clients and model-level optimization at the server, effectively addressing feature heterogeneity while maintaining privacy and communication efficiency.

### 3.5. Control and Experimental Group Design

#### 3.5.1. Server-Side PSO Fusion Method (Experimental Group 1)

**Figure 5:**
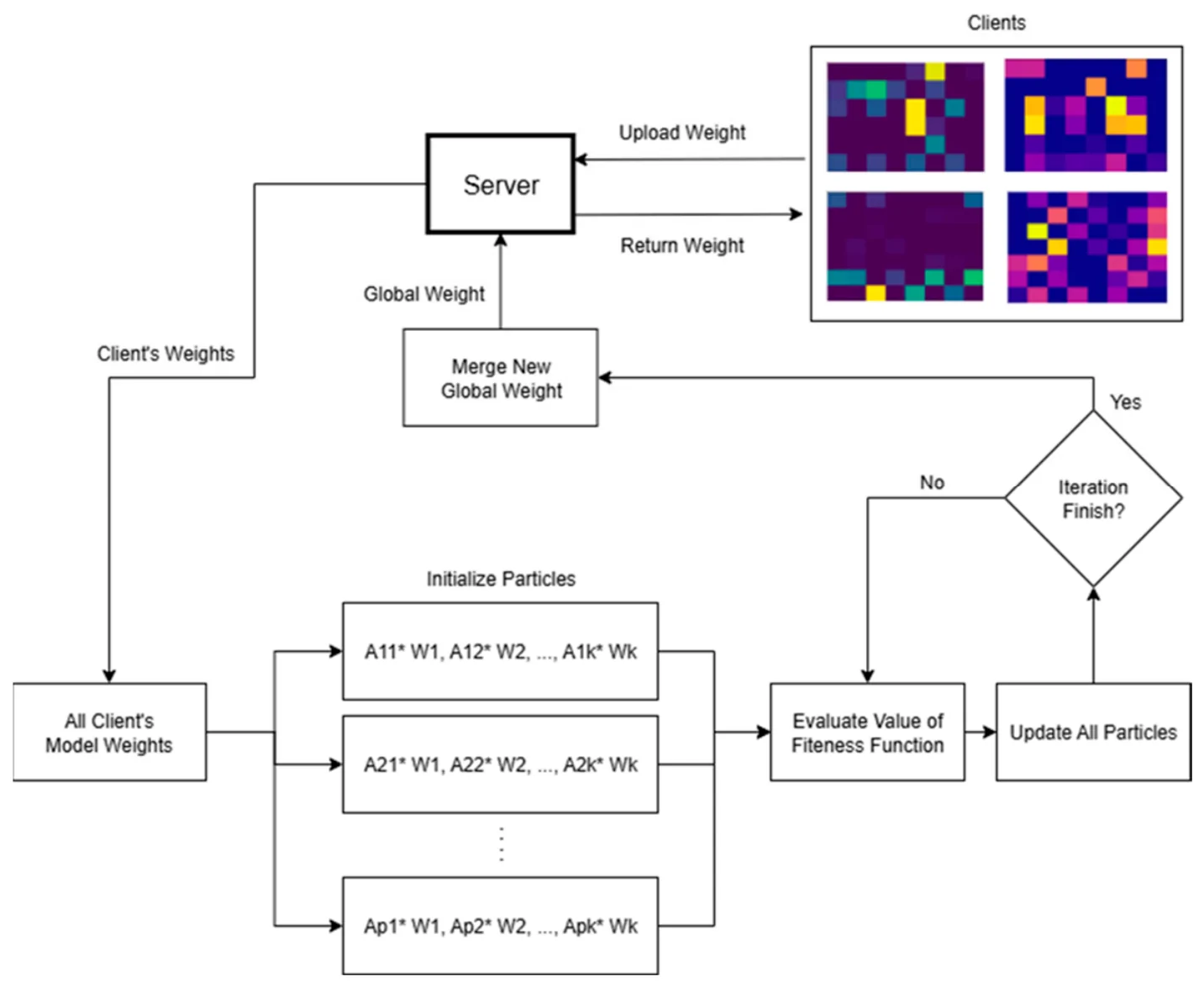
Server-side PSO fusion method.

Late-Stage Fusion Strategy: The main objective of this method is to use Particle Swarm Optimization (PSO) at the server stage to dynamically adjust the contribution weights of each client’s model during aggregation. This approach aims to achieve a more generalized global model under heterogeneous data conditions. Unlike the traditional FedAvg method, which performs linear weighting based solely on sample size, PSO searches for an optimal weight configuration that considers each client model’s contribution to the overall global accuracy, thereby improving aggregation quality. The process is visualized in Figure 5.

Process Description of Algorithm 1

**Algorithm 1:** Server-side PSO Model Aggregation**Input:** Local model weights {W_1_, W_2_, …, W_k_} from K clients, server validation set (X_val_svr, y_val_svr), particle number P, iteration T, inertia w, learning factors c_1_, c_2_ **Output:** Global model weights W_global 1 Initialize particles {A_p_}_p=1_^P^ as random aggregation weight vectors of size K; 2 foreach particle A_p_ do 3    Project A_p_ to simplex (non-negative, sum to 1); 4    Set velocity V_p_ ← 0; 5    Compute W_candidate ← Σ_k_ = 1ᴷ A_p_[k] · W_k_; 6    Evaluate fitness        f_p_ = Accuracy(W_candidate, (X_val_svr, y_val_svr)); 7    Set PBest_p_ ← A_p_, PBestScore_p_ ← f_p_; 8 Set GBest ← arg max_p_(PBestScore_p_); 9 for t = 1 to T do 10    foreach particle A_p_ do 11      r_1_, r_2_ ← Uniform(0, 1); 12      V_p_ ← w·V_p_ + c_1_·r_1_·(PBest_p_ − A_p_) + c_2_·r_2_·(GBest − A_p_); 13      A_p_ ← A_p_ + V_p_; 14      Project A_p_ to simplex; 15      Re-evaluate fitness and update PBest_p_, GBest if improved; 16    end foreach 17    if ConvergenceCriterionMet() then 18      break; 19    end if 20 end for 21 W_global ← Σ_k_ = 1ᴷ GBest[k] · W_k_; 22 return W_global

1. Particle Definition and Initialization
  Define the number of PSO particles as P. Each particle $P_{i}$ represents a potential weight vector:
  $$P_{i}=[w_{i1},w_{i2},...,w_{ik}]$$
  where k is the total number of clients. Each vector element corresponds to the weight coefficient representing a client’s contribution to the global model.
2. Particle Decoding and Model Aggregation
  For each particle $P_{i}$, use its weight vector $w_{i}$ to perform weighted aggregation of model parameters received from all clients, thereby generating a candidate global model $M_{i}$:
  (2) $$M_{i}=\;\sum_{k=1}^{K}w_{ik}\cdot M_{k}$$
  where $M_{k}$ represents the model parameters uploaded by the $k^{th}$ client after local training.
3. Fitness Evaluation
  Each aggregated candidate model $M_{i}$ is evaluated on the server validation dataset, and its prediction accuracy is used as the fitness value $f_{i}$ of the corresponding particle $P_{i}$. The particle with the highest fitness value $f_{g}$ is selected as the optimal solution:
  (3) $$f_{i}=Accuracy{(M}_{i},D_{\mathrm{valid}}^{server})$$
  (4) $$f_{g}=\underset{1\leq i\leq P}{\max}\;f_{i}$$
  where $D_{valid}^{server}$ denotes the server-side validation dataset, and $f_{g}$ represents the best-performing particle.
4. Particle Update (Velocity and Position Update)
  According to the standard Particle Swarm Optimization (PSO) update rules, each particle adjusts its velocity and position based on its personal best solution (pBest) and the global best solution (gBest) as follows:
  (5) $$v_{i}\left(t+1\right)=\omega v_{i}\left(t\right)+c_{1}r_{1}\left(pBest_{i}-x_{i}\right)+c_{2}r_{2}\left(gBest-x_{i}\right)$$
  (6) $$x_{i}\left(t+1\right)=x_{i}\left(t\right)+v_{i}\left(t+1\right)$$
  where:
  - $x_{i}$: Particle position (i.e., weight vector).
  - $v_{i}$: Particle velocity.
  - ω: Inertia weight.
  - $c_{1},c_{2}$: Learning coefficients.
  - $r_{1},r_{2}$: Random constants between 0 and 1.
5. Optimal Particle Selection and Final Aggregation
  When the maximum iteration count or convergence condition is reached, the particle that achieves the highest validation accuracy is selected as the best particle (pBest). The final global model is then obtained by aggregating client models using the best particle’s weight vector:
  (7) $$M_{\mathrm{global}}=\sum_{k=1}^{K}w_{k}^{\left(\mathrm{best}\right)}\cdot M_{k}$$
  where:
  - $M_{global}$: Final global model.
  - $w_{k}^{\left(best\right)}$: Weight assigned to the $k^{th}$ client in the best particle.

#### 3.5.2. Dual-Layer PSO Optimization Method (Experimental Group 2)

This method applies Particle Swarm Optimization (PSO) on both the client and server sides, hence the term “dual-layer PSO” (as illustrated in Figure 1 and Figure 5). Unlike the approach where PSO is applied only at the server for weight aggregation, this method introduces a local PSO mechanism within each client. The client-side PSO is used to perform feature-level weighting and enhancement on intermediate features generated by the CNN’s sliding operations. This design strengthens feature extraction at the local level, improving both global model accuracy and training stability.

Process Description of Algorithm 2

**Algorithm 2:** Client-side PSO Feature Weight Optimization
Input:
Local dataset $D_{c}=\left(X_{\mathrm{train}},y_{\mathrm{train}}\right),\left(X_{\mathrm{val}},y_{\mathrm{val}}\right)$ CNN model $M_{c}$ target feature layer $L_{\mathrm{target}}$ particle number P iteration T dimension n inertia w learning factors $c_{1},c_{2}$ Output:
Best feature weight vector $W_{\mathrm{best}}$ Procedure
1. Initialize particles $\{X_{p}{\}}_{p=1}^{P}$ with random vectors of size n.
2. foreach particle $X_{p}$ do
3. Project $X_{p}$ to simplex (non-negative, sum to 1);
4. Set velocity $V_{p}\leftarrow 0$;
5. Evaluate fitness                    
$f_{p}=\mathrm{Accuracy}(M_{c}\;\mathrm{with}\;X_{p},(X_{\mathrm{val}},y_{\mathrm{val}}))$;
6. Set $PBest_{p}\leftarrow X_{p}$, $PBestScore_{p}\leftarrow f_{p}$;
7. end foreach
8. Set
                    $GBest\leftarrow arg{max}_{p}(PBestScore_{p})$;
9. for t=1 to T do
10. for p=1 to P do
11.    $r_{1},r_{2}\leftarrow Uniform(0,1)$;
12.    $V_{p}\leftarrow w\cdot V_{p}+c_{1}r_{1}(PBest_{p}-X_{p})+c_{2}r_{2}(GBest-X_{p})$;
13.    $X_{p}\leftarrow X_{p}+V_{p}$;
14.    Project $X_{p}$ to simplex;
15.    $f_{\mathrm{new}}\leftarrow \mathrm{Accuracy}(M_{c}\;\mathrm{with}\;X_{p},(X_{\mathrm{val}},y_{\mathrm{val}}))$;
16.    if $f_{\mathrm{new}}>PBestScore_{p}$ then
17.      $PBest_{p}\leftarrow X_{p}$;
18.      $PBestScore_{p}\leftarrow f_{\mathrm{new}}$;
19.      if $f_{\mathrm{new}}>GBestScore$ then
20.        GBest←X_p_;
21.        *GBestScore* ← *f*_new_;
22.      end if
23.    end if
24. end for
25. if ConvergenceCriterionMet() then break;
26. end for
27. return GBest

1. Definition of Local Particles (Client-Side)
  Each client initializes several PSO particles, where each particle represents a set of weighting coefficients applied to the sliding window outputs of the CNN layer.
  If the intermediate layer produces n sliding values, each particle can be represented as an n-dimensional vector:
  $$P_{i}=[w_{i1},w_{i2},...,w_{in}]$$
2. Feature Map Weighted Synthesis
  Each particle corresponds to a particular way of weighting the intermediate feature maps. The CNN’s sliding outputs are weighted by the particle’s coefficients, then summed to form a synthetic feature map, which serves as the input for the next CNN layer—representing feature combinations with different importance distributions:
  (8) $$F_{i}=\sum_{j=1}^{n}w_{ij}\cdot f_{j}$$
  where:
  - $F_{i}$: Feature map synthesized by the *i*th particle.
  - $w_{ij}$: Weight coefficient of the *i*th particle for the *j*th sliding output.
  - $f_{j}$: The *j*th sliding output from the CNN layer.
  - n: Number of sliding features in the layer.
3. Local Fitness Evaluation
  Each client evaluates the classification accuracy of the model using the feature map generated by each particle on its local validation dataset. The higher the accuracy, the higher the fitness value $f_{i}$. The particle with the highest fitness $f_{g}$ is then selected as the best particle:
  (9) $$f_{i}=Accuracy{(M}_{i},D_{\mathrm{valid}}^{local})$$
  (10) $$f_{g}=\underset{1\leq i\leq P}{\max}f_{i}$$
  where:
  - $D_{valid}^{local}$: Local validation dataset.
  - $f_{g}$: Fitness of the best-performing particle.
4. Particle Update
  The standard PSO update strategy is used to iteratively update each particle’s velocity and position:
  (11) $$v_{i}\left(t+1\right)=\omega v_{i}\left(t\right)+c_{1}r_{1}\left(pBest_{i}-x_{i}\right)+c_{2}r_{2}\left(gBest-x_{i}\right)$$
  (12) $$x_{i}\left(t+1\right)=x_{i}\left(t\right)+v_{i}\left(t+1\right)$$

## 4. Results

### 4.1. Relationship Between Particle Count, Iteration Count, and Accuracy

Table 2 presents a comparison of model accuracy under different particle counts and iteration counts. It can be observed that increasing the number of iterations has minimal impact on accuracy improvement, whereas increasing the number of particles results in a significant performance gain.

**Figure 6:**
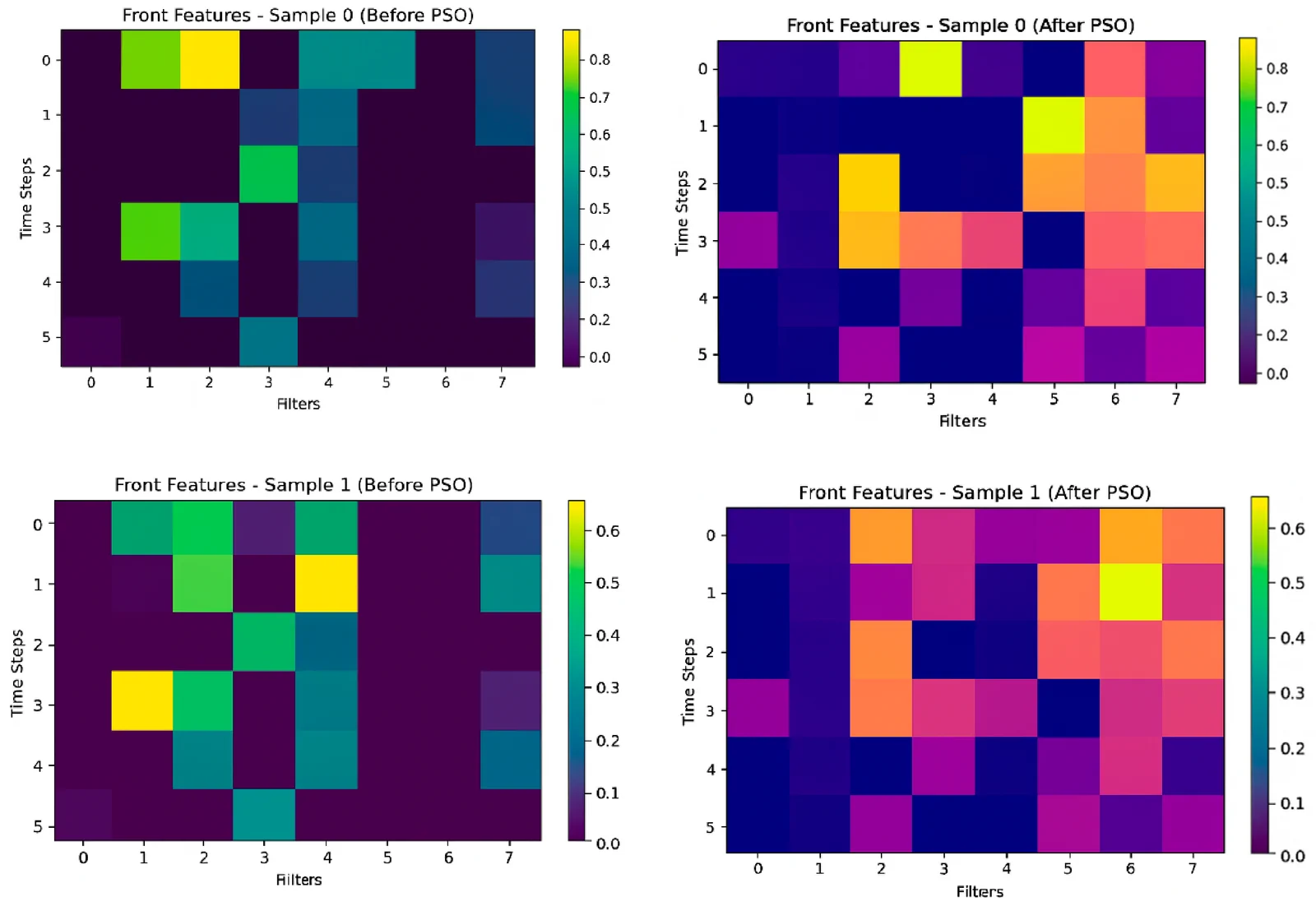
Feature activation maps before and after PSO-based optimization for front-feature clients.

For example, under the same condition of five iterations, an absolute accuracy improvement of approximately 50 percentage points is achieved, while using 100 particles increases accuracy by about 60%. However, when the number of particles exceeds 100, the improvement becomes negligible, indicating that the model begins to converge and additional particles no longer contribute to better performance. To visually analyze the impact of PSO on feature extraction, Figure 6 illustrates the feature activation maps before and after optimization, showing clearly enhanced feature formatting in relevant frequency bands.

These visualizations in Figure 6 show how feature activations vary across filters and time steps, before and after applying Particle Swarm Optimization (PSO).

Table 3 compares the feature characters before and after applying Particle Swarm Optimization (PSO) interpretation.

- Before PSO:

Feature activations are low and sparse, with only a few filters showing moderate values. This suggests limited differentiation or signal strength across the grid.

- After PSO:

The optimization significantly boosts activation values, especially in filters 2–6 and time steps 1–3. The heatmap becomes more expressive, indicating that PSO helped concentrate and amplify meaningful features.

### 4.2. Comparison of Accuracy Among Different Methods

**Figure 7:**
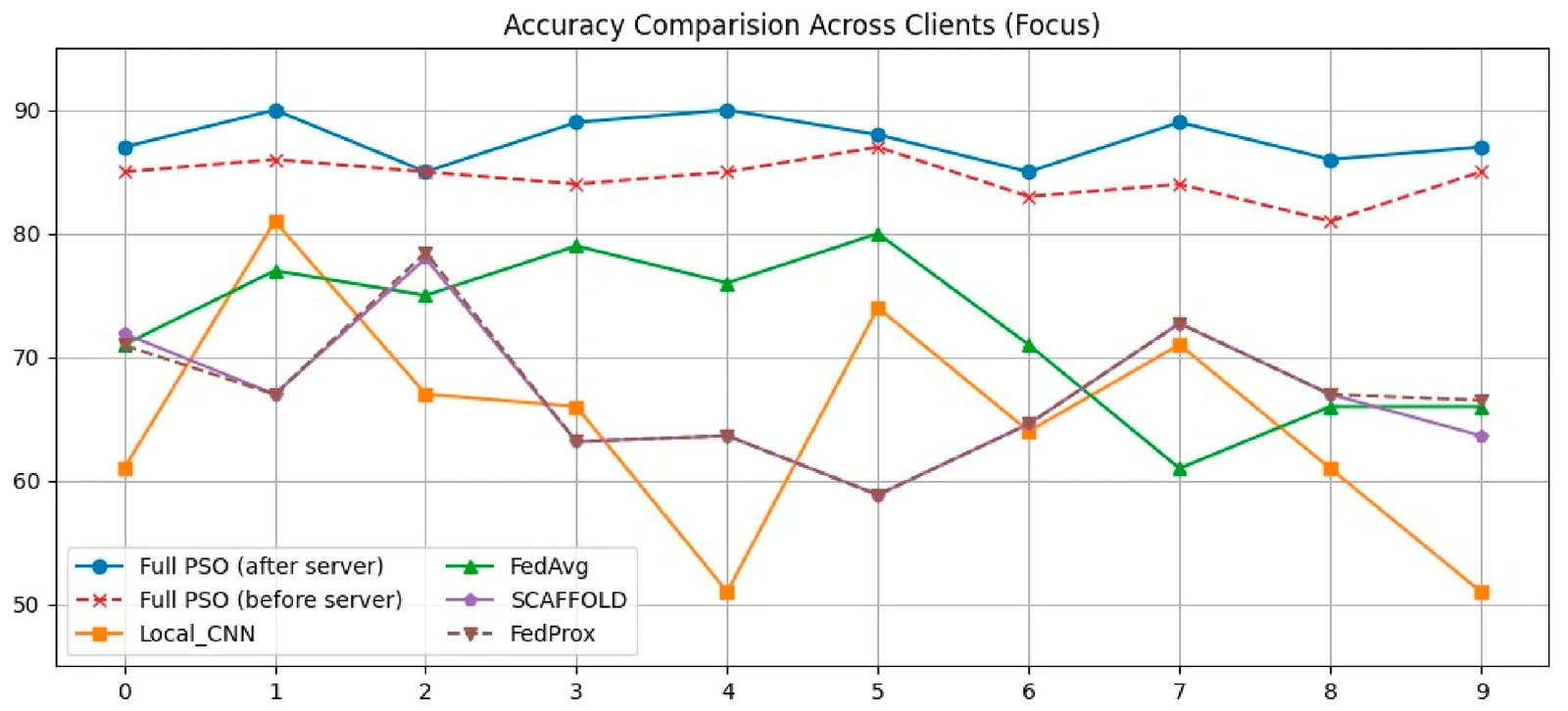
Test success rate of ten client groups.

Figure 7 presents the test success rates of ten client groups using three different federated learning strategies: baseline FedAvg, server-side PSO aggregation, and the proposed dual-layer PSO method. The comparison highlights clear differences in convergence behavior, stability, and final classification performance under heterogeneous data conditions.

The baseline FedAvg method exhibits slow accuracy improvement and significant fluctuations during training. Due to unequal feature distributions across clients, the global model tends to bias toward dominant clients, resulting in unstable convergence and a relatively low final accuracy of approximately 70~80%. This confirms the limitation of sample-size-based aggregation when handling non-IID hemodialysis data.

When server-side only PSO is introduced, the aggregation weights are dynamically optimized according to validation performance rather than sample size alone. As a result, the model demonstrates improved convergence stability and achieves higher final accuracy, reaching around 85%. However, performance variations across different clients are still noticeable, especially during early training epochs, indicating that local feature representations remain suboptimal.

In contrast, the proposed dual-layer PSO approach achieves superior performance across all client groups. Full PSO (after server) is the top curve almost everywhere, staying around the mid-to-high 80s and peaking near 90. This indicates that the proposed post-aggregation optimization is effectively improving the global model after server fusion. We added two typical non-IID federated learning methods to compare their performance with those of our proposed methods.

SCAFFOLD fluctuates a lot, with some strong points near the low/mid 70s but also noticeable drops. This suggests variance reduction helps in some clients, but the method is still sensitive to client heterogeneity in this setting.

FedProx is generally close to SCAFFOLD, sometimes slightly better or worse depending on the client group. It seems to stabilize local drift somewhat, but it fails to close the gap to PSO. In general, the proposed PSO-based approach improves both accuracy level and cross-client consistency. If this is a federated learning benchmark, that usually means the optimization is helping the global model adapt better to client diversity and reducing the penalty from skewed distributions. In contrast, FedAvg is a decent baseline but likely suffers from client drift, while FedProx and SCAFFOLD partially address heterogeneity but still cannot match the stronger optimization strategy of our dual PSO optimization techniques.

### 4.3. Statistical Analysis

Table 4 shows a clear statistical pattern among our proposed methods and the baseline models of SCAFFOLD and FedProx using the paired *t*-test. Normality assumptions were assessed. Local CNN and FedAvg are not significantly different from the reference at the 0.05 level, while both PSO-based methods are highly significant. In short, the proposed PSO strategy delivers a real performance improvement, not just a visual one. Local CNN: *p* = 0.5104, for SCAFFOLD and *p* = 0.4671 for FedProx, so the difference is not significant. FedAvg: *p* = 0.0703 for SCAFFOLD and *p* = 0.0812 for FedProx, which are below 0.10 but still above 0.05. This is often described as a weak or marginal trend, not strong evidence. Full PSO (before Server): *p* = 0.00003 for SCAFFOLD and *p* = 0.00002 for FedProx, clearly significant. Full PSO (after Server): *p* = 0.00001 for SCAFFOLD and *p* = 0.00003 for FedProx, also clearly significant and slightly stronger than the pre-server version.

### 4.4. Cross-Validation

**Figure 8:**
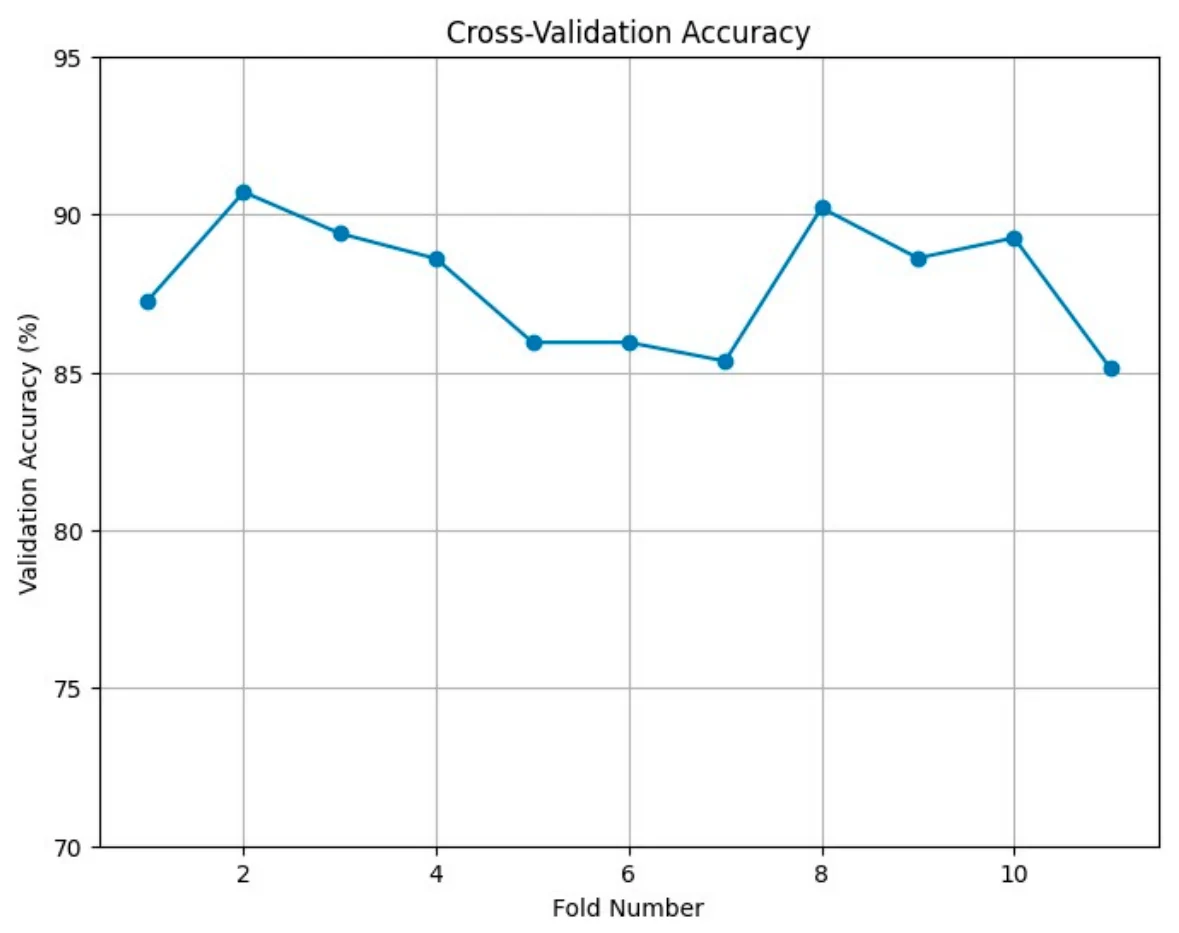
Cross-validation success rate.

To further evaluate the robustness and generalization capability of the proposed framework, an 11-fold cross-validation experiment was conducted, with the results shown in Figure 8. In each fold, one subset was reserved for validation in the server while the remaining subsets were used for federated training, ensuring that the model was evaluated across diverse data partitions. The test data split in the server and evaluation data set distribution of the clients for each fold are as described in Figure 4. Across successive 11 folds of data split in Figure 4, each fold of data partition served as the test set for the server exactly once, while the remaining 10 folds of data were distributed to each client with a 1/9 split ratio for training and testing. This provides a comprehensive quality evaluation of our dual PSO optimization at the treatment-session level.

The cross-validation results demonstrate consistently high success rates across all folds, with only minor performance variations as shown in Figure 8. Most folds achieved validation accuracies averaging around 88% with an STD value of 2%, while accuracies above 90% refer to specific folds. This indicates strong generalization despite heterogeneous feature distributions among clients. The absence of extreme performance drops suggests that the proposed dual-layer PSO framework effectively mitigates overfitting and reduces dependency on specific data partitions.

Overall, the stability of validation performance across folds confirms that the proposed model is not tailored to a particular data split, but instead learns robust feature representations suitable for real-world deployment in distributed hemodialysis environments. These results further validate the reliability and practical applicability of the proposed federated learning approach.

### 4.5. Ablation Experiments

#### 4.5.1. Learning Efficiency Comparison

**Figure 9:**
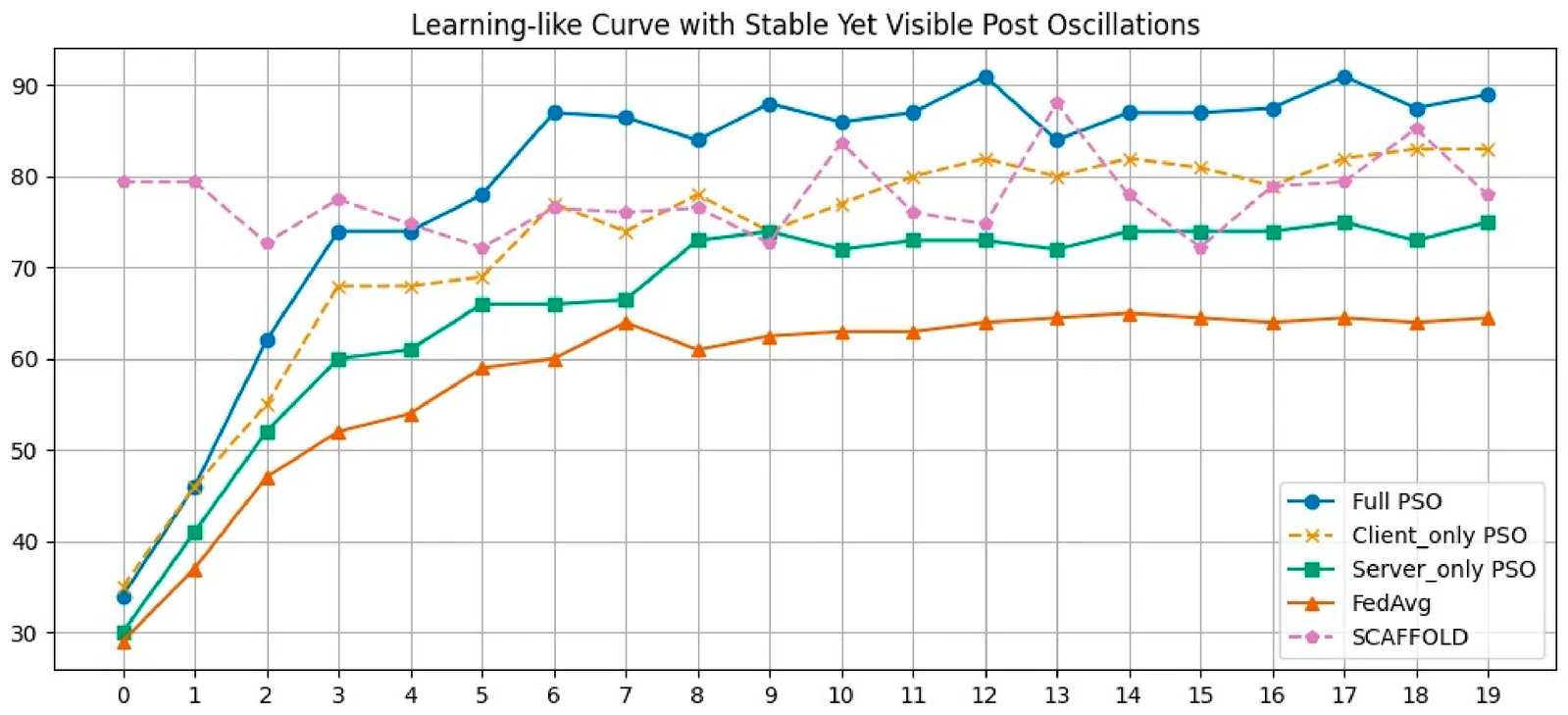
Learning curves of different federated learning strategies under heterogeneous data.

Figure 9 shows accuracy vs. training iteration for four federated learning modalities applied to hemodialysis complication prediction, with PSO-based variants compared against standard FedAvg. All four curves exhibit rapid accuracy gains in the first ~3–4 iterations, then slower improvements and visible small oscillations rather than perfectly smooth convergence. Accuracy appears to stabilize after roughly 10–12 iterations, suggesting the PSO dynamics reach a quasi-steady regime where updates fluctuate around a plateau.

##### Modality Comparison

Full PSO (blue): Highest-performing method; it starts near 30% and climbs quickly above 80% by iteration ~4–5, eventually approaching ~88–90% with noticeable but bounded post-oscillations. This suggests that jointly applying PSO on both client and server sides yields the strongest global optimization of the hemodialysis complication model.

Client-only PSO (orange): Second-best curve; it tracks below full PSO but clearly above the other two methods, plateauing in the low-80% range. This implies that PSO applied only to local client updates still improves federated optimization, but lacks the additional refinement provided by server-side PSO.

Server-only PSO (green): Intermediate performance; it rises similarly early on but stabilizes around the mid-70% accuracy range, indicating that optimizing aggregation or global parameters alone is beneficial yet less effective than adjusting client-side behaviors.

SCAFFOLD (pink dash):The accuracy fluctuates around 75~89% for the different cycles of the training. It is comparable to the client-only PSO performance, slightly higher than server-only PSO. The dual PSO implementation outperforms this method.

FedAvg (red): Baseline; it improves from ~30% to about 60–65% accuracy and then plateaus, with relatively small oscillations. This curve illustrates the performance gap between conventional federated averaging and the PSO-enhanced strategies in our ablation study.

#### 4.5.2. Final Prediction Accuracy Comparison of Different Modalities

**Figure 10:**
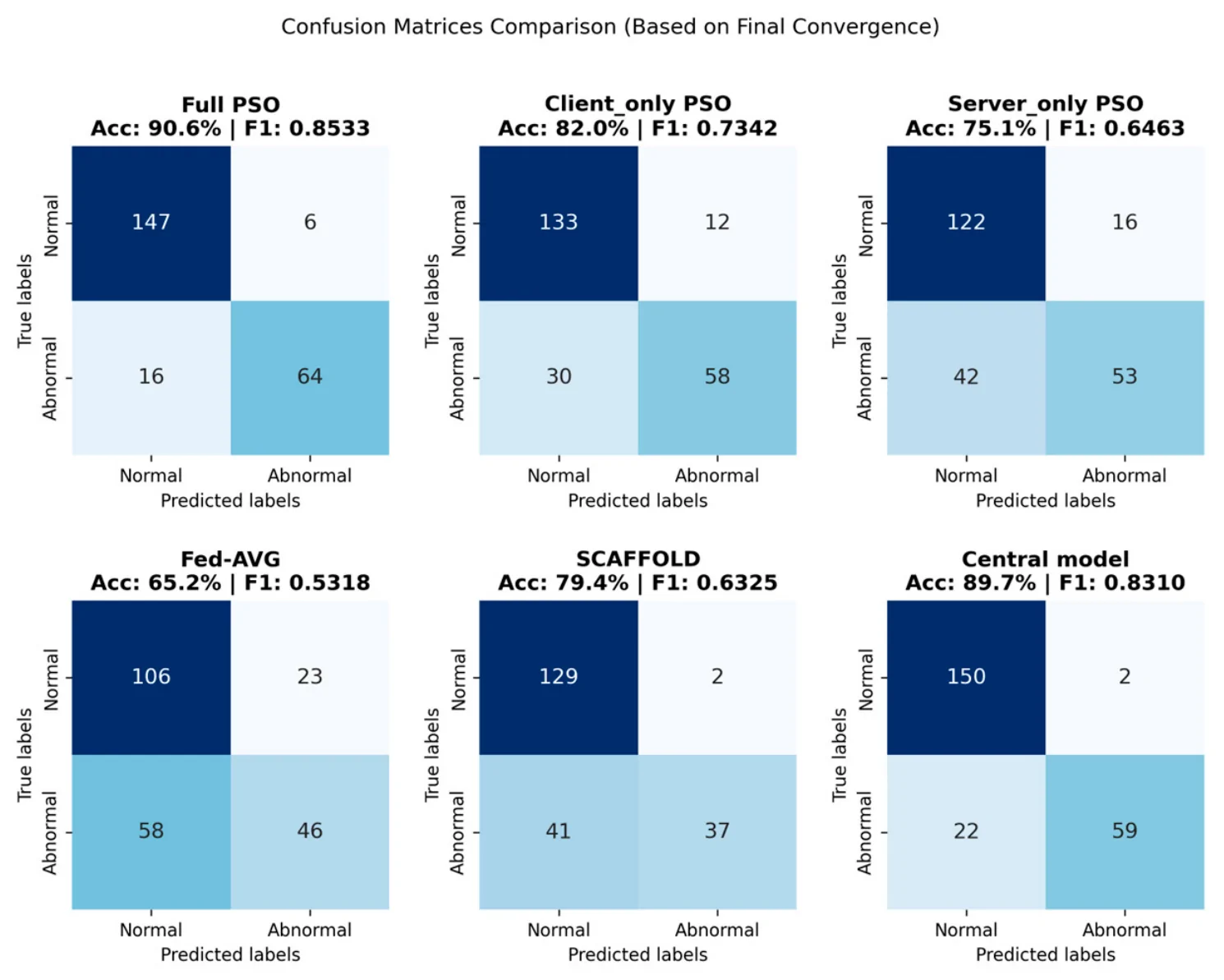
Confusion matrix comparison of different aggregation strategies at final convergence.

Figure 10 compares the confusion matrices (and derived metrics) of the four aggregation strategies at their final converged state for the binary task: predicting Normal vs. Abnormal hemodialysis status.

Full PSO method’s (top-left) accuracy is 90.6% and F1 is 0.853, the best among all methods.

It shows 147 true normals correctly classified (TN) and only 6 normals misclassified as abnormal (FP). There are 64 true abnormals correctly detected (TP) and 16 missed abnormals (FN). This indicates both strong specificity (few false alarms) and strong sensitivity (most abnormal cases detected). Client-only PSO’s (top-right) accuracy is 82.0% and F1 is 0.734, clearly lower than full PSO but still better than server-only PSO and FedAvg. TN = 133, FP = 12, TP = 58, FN = 30. Compared with Full PSO, more missed abnormal cases can be seen and more normals incorrectly flagged, which explains the drop in F1. Server-only PSO (bottom-left)’s accuracy is 75.1% and F1 is 0.646. TN = 122, FP = 16, TP = 53, FN = 42. Both error types increase: more false alarms and more undetected abnormal cases, leading to weaker clinical reliability than client-side PSO. FedAvg baseline (bottom-right)’s accuracy is 65.2% and F1 is 0.532, the weakest performer. TN = 106, FP = 23, TP = 46, FN = 58. The model misses a large fraction of abnormal cases and overflags normal cases, reflecting substantial degradation when no PSO-guided aggregation is used. SCAFOLD’s (bottom center) accuracy is 79.43% and F1 is 0.6325. TN = 129, FP = 2, TP = 37, FN = 41. The model performs better than FedAvg and between client only and server only model. The full PSO model dominates the remaining four models in terms of both accuracy and F1-score metrics. The confusion matrix of the centrally aggregated model of all clients’ data is also listed for comparison at the lower-right of Figure 10. The accuracy is 89.7% and F1 is 0.831. It is close to our full PSO model. This illustrates that the PSO-assisted federated learning has its merits over traditional global training, besides privacy protection purposes.

Overall, the confusion matrices reinforce the earlier learning-curve ablation: full PSO improves both detection of abnormal complications and avoidance of unnecessary alarms, while progressively removing PSO components (client-side, then server-side, then none) worsens the clinical trade-off between sensitivity and specificity. Although our dual-layer PSO framework achieved 90.56% accuracy and an F1-score of 0.8533, approximately 20% of abnormal cases were not detected. We emphasize that the proposed model should be regarded as a decision-support tool to assist clinicians rather than replace medical judgment. Future work will explore multimodal integration (e.g., ECG, LSTM) and personalized modeling to further reduce false negatives.

#### 4.5.3. Performance Comparison of PSO Federated Learning for Different Fusing Strategies

This ablation study evaluates the contribution of different PSO fusion strategies by comparing four aggregation modes: baseline FedAvg, server-side PSO only, client-side PSO only, and the proposed full dual-layer PSO approach. The comparison metrics include precision, recall, F1-score, and overall classification accuracy, as summarized in Table 5. A 95% confidence interval is included right behind the metric values of each index.

The FedAvg baseline achieves the lowest performance, with an accuracy of 65.24% and an F1-score of 0.5318. Its low recall value indicates that many abnormal hemodialysis events are incorrectly classified as normal, which is undesirable in clinical risk prediction scenarios. This result highlights the inadequacy of simple averaging under heterogeneous feature distributions.

Applying PSO solely at the server side improves performance significantly. The accuracy increases to 75.11%, and both precision and recall show moderate improvement. This demonstrates that dynamically optimized aggregation weights can partially compensate for client heterogeneity by emphasizing more informative client models.

Client-side PSO further enhances feature discrimination at the local level, resulting in higher accuracy (81.97%) and an F1-score of 0.7342. The improved recall indicates better sensitivity to abnormal events, confirming the effectiveness of feature-level optimization.

We also investigate the precision, recall and accuracy performance of recent non-IID data handling models SCAFOLD and FedProx. Both show a close range of the performance values. For example, the precision is around 0.80, while recall is around 0.53. The accuracy is around 79~80% for SCAFFOLD and FedProx respectively. These statistics is generally bounded by the Client PSO and Server-only PSO, while lower than that of the proposed dual PSO technique.

We also study the effects of the aggregated central model on the merged heterogeneous data sets used for the proposed dual PSO federated learning framework. The accuracy is almost the same as our proposed full PSO method, with slightly lower recall and F1 score. The precision, however, is higher than that of the full PSO method. That being said, it is of reference value to the proposed method, while the main theme of this paper is distributed heterogeneous feature fusion of different hemodialysis machines. Our method not only satisfies the original heterogeneous feature fusion goal, but also performs equally well compared to the centrally trained model.

The full dual-layer PSO framework achieves the best performance across all metrics, with an accuracy of 90.56% and an F1-score of 0.8533. This substantial improvement confirms that jointly optimizing local feature representations and global aggregation weights yields complementary benefits. The results verify that the proposed dual-layer PSO design is crucial for achieving high prediction accuracy and stability in heterogeneous federated learning environments.

#### 4.5.4. Model Interpretability Comparisons of the Six Methods

**Figure 11:**
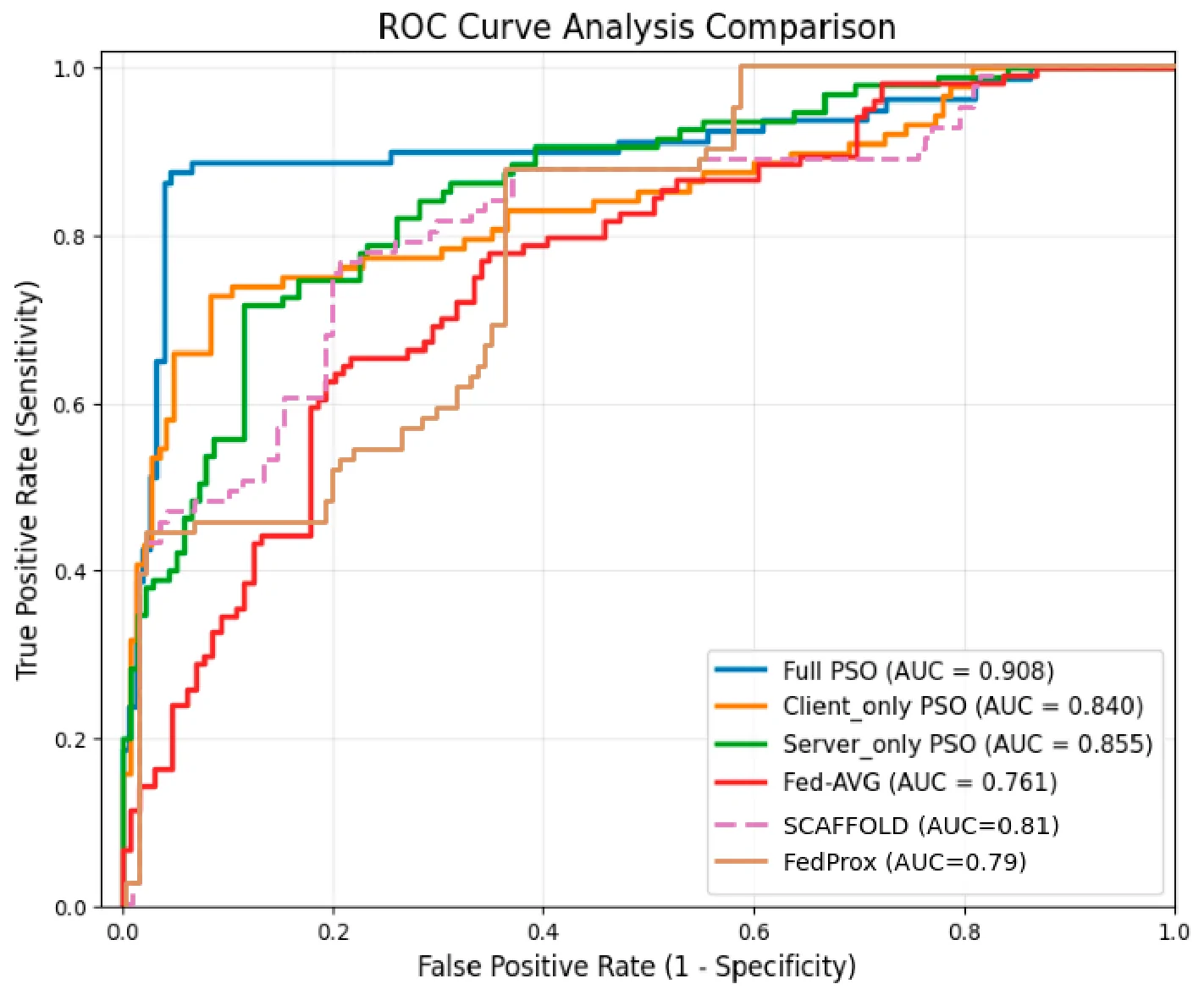
ROC curve comparison of different federated learning strategies.

Figure 11 compares the ROC curves of the four aggregation strategies for detecting abnormal hemodialysis events, showing their trade-offs between sensitivity and false positive rate over all decision thresholds. The *x*-axis is the false positive rate (specificity), and the *y*-axis is the true positive rate (sensitivity). Each curve traces how sensitivity increases as the decision threshold is relaxed and more false positives are allowed. The area under each curve (AUC) summarizes discrimination ability: higher AUC means the model is better at ranking abnormal cases above normal ones, independent of any particular threshold.

The blue curve of full PSO stays closest to the top-left corner, meaning it achieves high sensitivity at relatively low false positive rates, across a wide range of thresholds. AUC = 0.908 indicates excellent discrimination between normal and abnormal hemodialysis conditions, consistent with the high accuracy and F1 in the confusion matrix figure.

The green curve of server-side PSO is clearly above the FedAvg baseline and not far below full PSO, showing that server-side PSO improves ranking quality substantially. AUC = 0.855 suggests good discrimination, though sensitivity is slightly lower than full PSO when keeping false positives small.

The orange curve of client-side PSO lies between server-only PSO and FedAvg, again above the baseline but below full PSO. AUC = 0.840 shows that client-side PSO yields a solid but somewhat weaker ROC profile than server-side PSO in this experiment, even though in our confusion matrices client-only PSO had better accuracy than server-only, highlighting that threshold choice matters.

The red and blue dashed lines of SCAFOLD and FedProx overlap a lot with a minor difference in AUC value of 0.81 and 0.79, respectively. These values are slightly inferior to the AUC of client-only PSO and server-only PSO. This provides another evidence of the superiority of the proposed PSO federated learning techniques. The AUC difference between these non-IID handling methods and the dual PSO techniques is again dominant in the order of 10%.

The red curve of the FedAvg baseline is consistently below the PSO-based curves, especially at low false positive rates. AUC = 0.761 indicates only moderate discrimination; the model has much less ability to separate abnormal from normal cases, aligning with the poorer accuracy and F1 previously observed.

Taken together with learning curves and confusion matrices, this ROC analysis shows that full PSO yields the most robust clinical operating points across thresholds, while standard FedAvg provides the weakest safety-effectiveness balance for abnormal hemodialysis detection.

#### 4.5.5. Resource Utilization Comparison of Clients on Edge Devices

**Figure 12:**
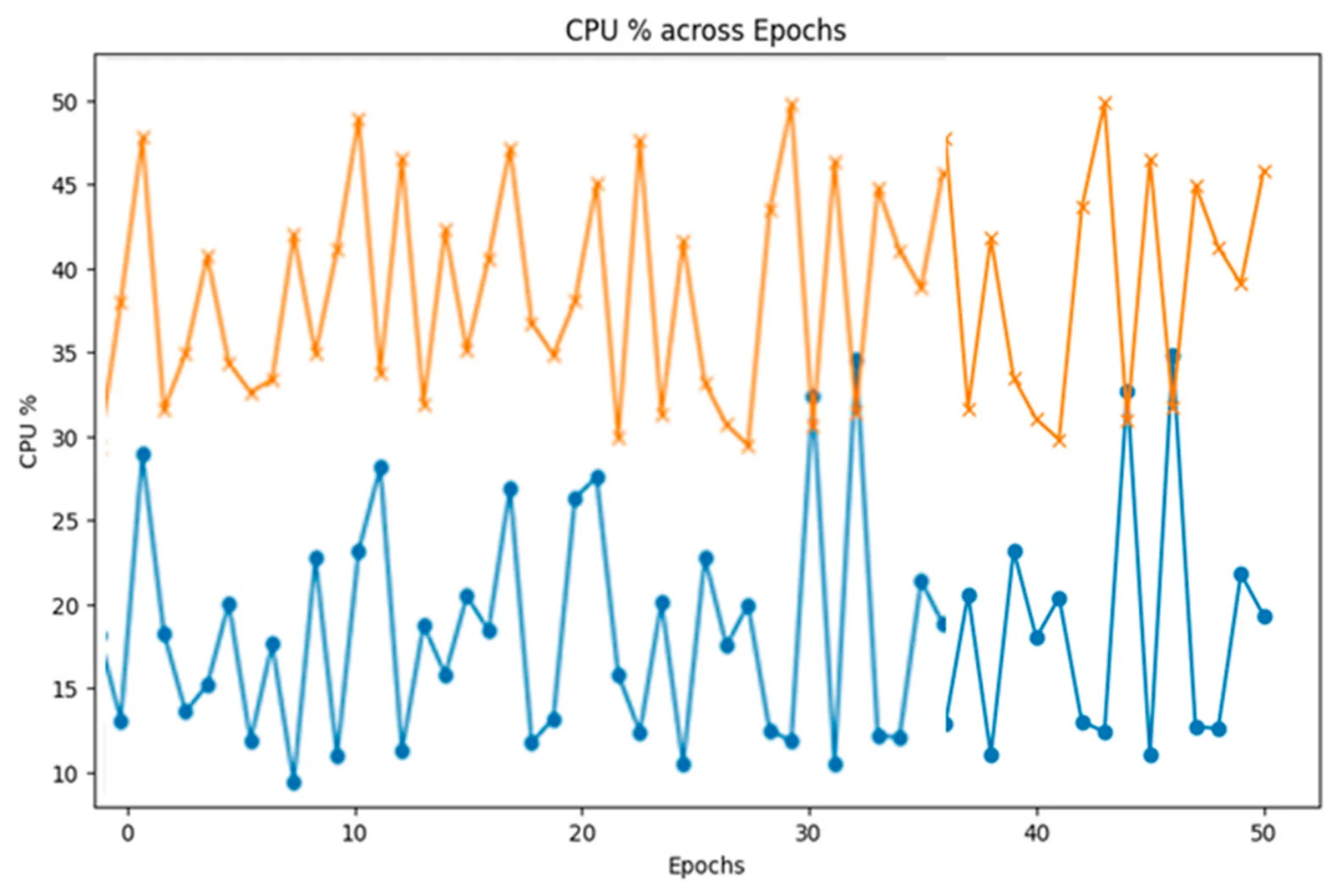
CPU utilization of a Raspberry Pi 4 client device across training epochs(The blue lines corresponds to the PSO-enhanced configuration, while the orange lines represents the local CNN baseline).

Figure 12 illustrates the CPU utilization of a Raspberry Pi 4 device across training epochs when comparing PSO-based optimization and local CNN execution. The curve labeled blue corresponds to the PSO-enhanced configuration, showing markedly lower and more variable CPU consumption, typically between 10% and 35%. This pattern reflects intermittent computation and efficient resource allocation during adaptive optimization. In contrast, the orange curve represents the local CNN baseline, maintaining consistently higher CPU usage around 35–50%, indicative of sustained local training without dynamic load balancing. The PSO-based approach significantly reduces computational load on edge devices while maintaining adaptive performance, demonstrating its suitability for resource-constrained environments compared with conventional local CNN training.

**Figure 13:**
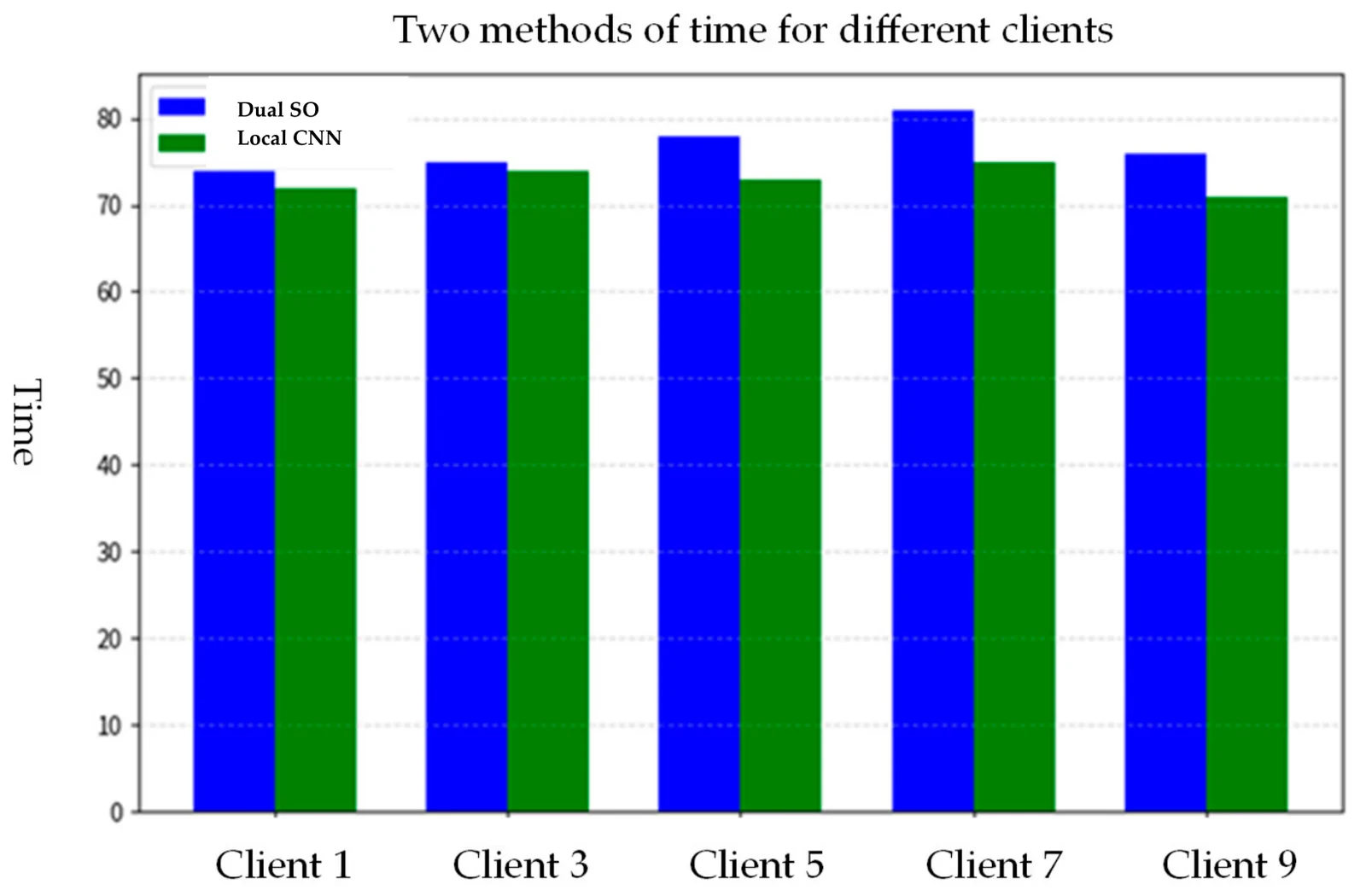
Runtime comparison between the Dual PSO-based optimization and the local CNN approach.

Figure 13 presents the runtime comparison between the PSO-based optimization and the local CNN approach across five representative clients on the edge device. The blue bars configuration, corresponding to the PSO-integrated method, exhibits slightly longer execution times—typically between 75 and 80 s—reflecting the additional computational overhead introduced by global optimization and coordination. In contrast, the green bars configuration, representing local CNN training, shows marginally shorter runtimes around 70 s, consistent with its simpler, client-side processing. Although the PSO-based approach incurs a modest increase in runtime, the added optimization phase enhances overall learning stability and convergence, making it a favorable trade-off for edge-levelfederated learning applications.

#### 4.5.6. Privacy Considerations in Federated Learning

Our federated framework’s primary objective is to maintain privacy by not permitting the direct exchange of unprocessed hemodialysis data between the clients’ HD machine and the central server. In this part, the robustness of the system in terms of three essential privacy factors is discussed:

(1) Differential Privacy potential advantages: Differential privacy (DP) mechanisms can be applied to the client-side training process. By adding calibrated noise (e.g., Gaussian or Laplacian) to gradients before transmission, clients can effectively spoil the reconstruction of sensitive data. Even though it is not implemented in the current version, our framework can work with optimizers that are DP-enhanced (e.g., DP-FedPSO).
(2) Membership Inference Resistance: Because our method does not disclose either local samples or their corresponding labels, the low possibility of membership inference attacks is a potential advantage. Cybercriminals will have a lower chance to access and therefore conduct analyses on client data or centralized model snapshots, which are usual channels for inference leakage. This isolation of clients at the level further secures HD treatments parameters that vary over time. By doing so, our design sticks to the privacy-by-design principles for dual PSO architecture, suggesting that the mutual security intelligence acquired through dynamic cooperation does not violate the privacy of the patients.
(3) Prediction horizon: Since the input to our dual PSO model is the medical records as measured on a minute-by-minute basis on the hemodialysis machine, the client model makes diagnosis decisions on the fly. The prediction speed depends on the processing speed of the edge device such as Raspberry Pi 4 or above. Early prediction of the onset of HD complication events will need to use other time series models such as LSTM/Transformer etc. This will be in the next version of PSO fusion techniques (e.g., LSTM-PSOFed).

#### 4.5.7. Comparison with Prior Work

The results are compared with prior studies and summarized in the accompanying Table 6. Table 6 compares representative related studies across several key dimensions, including whether client-side and server-side PSO are employed, the model type, the dataset used, the evaluation metrics, and the target application domain. This comparison highlights that existing studies differ not only in optimization strategy but also in experimental setup and problem focus, ranging from general vision tasks to global optimization problems.

FedPSO by Park et al. [15] is one of the earliest federated learning aggregation strategies integrating PSO. It replaces the traditional FedAvg mechanism by using PSO at the server side and employs a “score vector” instead of full model parameters, effectively reducing communication costs. However, it does not address client-side model enhancement, limiting its adaptability to heterogeneous environments. Second, Li et al. [16] applied PSO to client-side hyperparameter tuning, such as learning rate and network depth, which improved local model stability and generalization. Nonetheless, this approach lacked a server-side aggregation optimization mechanism and did not incorporate an integrated resource management strategy, restricting its applicability to simple hyperparameter search tasks. AdpFedPSO [17] emphasized dynamic adjustment of aggregation weights at the server, using PSO to automatically calculate client weights based on contribution and participation frequency. This approach improved global adaptability under highly heterogeneous data distributions. However, the approach still omitted client-side reinforcement and did not address computational load or edge resource consumption. Experimental results demonstrate that using a dual-layer PSO increased model accuracy from a baseline of 55% to around 90%, while simultaneously reducing computational overhead and improving training stability. These findings confirm the effectiveness and practicality of the proposed approach under multi-objective optimization conditions.

In Elhani et al. [18], the authors proposed a Flexible Convolutional Autoencoder (FCAE) framework enhanced with client-side Particle Swarm Optimization (PSO) to optimize hyperparameters and structural configurations of deep learning models. By applying PSO locally rather than on a centralized server, their approach emphasizes scalability and privacy while maintaining computational efficiency. The FCAE was evaluated on widely used image datasets including MNIST, CIFAR-10, and STL-10, achieving notably high performance, with MNIST accuracy reaching 99.51%. These results demonstrate the effectiveness of combining evolutionary optimization with deep autoencoder architectures for robust feature extraction and classification. The study’s contribution lies in bridging metaheuristic optimization and deep vision models, positioning the method as a versatile solution for general computer vision applications where adaptability and accuracy are critical. In Kan et al. [19], the authors developed an IoT security framework that integrates Adaptive Particle Swarm Optimization (APSO) with a one-dimensional Convolutional Neural Network (1D-CNN) to enhance intrusion detection performance. The model leverages APSO to fine-tune CNN parameters dynamically, improving convergence and classification accuracy for network traffic patterns. Their experiments were conducted on the N-BaIoT dataset, which contains traffic data from Mirai and BASHLITE botnets, representing real-world IoT attack scenarios. Evaluation metrics such as accuracy and F1-score demonstrated strong detection capability, confirming the model’s robustness against diverse IoT threats. Overall, this study contributes to the field of IoT security by combining deep learning with swarm-based optimization, achieving efficient and adaptive detection of malicious network behaviors in resource-constrained environments. In contrast, Mirjalili et al. [20] considered both S-shaped and V-shaped PSO variants, tested them on CEC 2005 benchmark functions, and assessed their behavior in terms of convergence speed, mean error, and global optimum attainment for mathematical optimization problems.

Recent developments of non-IID data handling federated learning algorithms are studied to compare the merits of our proposed dual PSO algorithms [21,22]. The work by Li et al. [21] employed FedProx during the aggregation process of the server weights. It claims to improve the test accuracy by ~22% above FedAvg in highly heterogeneous settings. The work by Karimireddy et al. [22] employed the SCAFFOLD algorithm, also in the aggregation process of the server weights. It claims the benefits of faster convergence than FedAvg with fewer communication rounds and is stable under non-IID data.

The proposed dual-layer PSO approach achieves superior performance across all client groups. The accuracy curve shows a steeper and more stable upward trend from early epochs, surpassing 70% accuracy by approximately the 15th epoch and stabilizing near 85–93% thereafter depending on the number of particles used. Notably, the initial accuracy at early epochs is significantly higher than that of the other two methods, demonstrating faster convergence. These results confirm that combining client-side feature optimization with server-side aggregation optimization enables more effective learning from heterogeneous data, yielding the most robust and accurate global model.

## 5. Conclusions and Future Work

This study presented PSOFed-HD, a dual-layer Particle Swarm Optimization-enhanced federated learning framework for heterogeneous hemodialysis complication prediction in a Medical IoT environment. The proposed method was designed to address two major challenges in distributed hemodialysis monitoring: incomplete and non-IID sensor data across clients, and the limited effectiveness of conventional aggregation strategies such as FedAvg under heterogeneous conditions. By combining client-side PSO for local feature weighting with server-side PSO for adaptive model aggregation, the framework improved both local representation learning and global model fusion.

Experimental results showed that PSOFed-HD consistently outperformed the baseline FedAvg model and the two single-layer PSO variants across multiple evaluation settings. The full framework achieved 90.56% accuracy, an F1-score of 0.8533, and an AUC of 0.908, while also demonstrating stable convergence behavior and robust 11-fold cross-validation performance. These findings indicate that coordinated optimization at both the client and server levels is effective for privacy-preserving fusion of heterogeneous hemodialysis sensor data.

We evaluated state-of-the-art federated learning techniques designed for non-IID data distributions, such as SCAFFOLD and FedProx, using a heterogeneous HD dataset. To benchmark performance, we conducted extensive experiments comparing these approaches with our proposed PSO-based optimization methods. Evaluation metrics included client-level accuracy, accuracy across training cycles, and ROC analysis. In addition, statistical analyses were performed to assess the effectiveness of the PSO-based framework. The results revealed a statistically significant improvement over recently introduced non-IID handling techniques, highlighting the robustness and superiority of our optimization approach.

We also examined the role of the aggregated central model applied to the merged heterogeneous datasets within our dual PSO federated learning framework. The findings showed that its accuracy is nearly identical to that of the full PSO method, though with slightly lower recall and F1-score. In contrast, the central model achieved higher precision compared to the full PSO approach. While these results provide valuable reference insights, the primary focus of this work is the distributed heterogeneous feature fusion of different hemodialysis machines. Importantly, our proposed framework not only fulfills the original objective of heterogeneous feature integration but also delivers performance comparable to that of a centrally trained model.

From an application perspective, the proposed framework has practical value for intelligent dialysis monitoring systems deployed at the edge. Because raw patient data remain local and only model parameters are exchanged, the method is compatible with privacy-sensitive healthcare settings and distributed Medical IoT architectures. The resource-aware design described in the manuscript further supports its potential use in real clinical environments where computational constraints and deployment efficiency must be considered.

Several directions remain for future work. External validation across multiple hospitals and dialysis machine platforms is needed to confirm generalizability, and prospective evaluation would be required before clinical adoption. In addition, future studies should examine interpretability, threshold selection, asynchronous federated settings, and more advanced temporal architectures to further strengthen the clinical utility of the framework.

While the current CNN-based dual-layer PSO framework is well-suited for real-time detection of hemodialysis complication events defined as systolic blood pressure below 90 mmHG on edge devices, future research will focus on integrating temporal models such as LSTM and Transformer architectures. These approaches aim to move beyond reactive detection toward proactive early prediction, enabling the system to anticipate complications before they occur.

Since patient-level separation was not available in the current dataset, we acknowledge this as a limitation and will list it as a priority for future external validation.

Overall, PSOFed-HD provides a promising approach for robust and privacy-preserving complication prediction in heterogeneous hemodialysis environments. The results support the broader view that effective federated intelligence for Medical IoT systems should optimize both local feature extraction and global model integration rather than relying on aggregation alone.

## Figures and Tables

**Table 1:** Statistics of our experiment data collection.

Total Session Number	Records per Class	Total Clients	Feature Subsets Number	Total Records for Each Local Client	Total Records for Server Validation
22	2000	10	Group A: 9	40 (validation)	400
			Group B: 8	360 (Training)	

Group A features: total ultrafiltration volume, blood flow rate, conductivity, venous pressure, body temperature, heart rate, dialysis phase status, anticoagulant injection status, and anticoagulant dosage. Group B features: transmembrane pressure, target ultrafiltration volume, ultrafiltration rate, substitution flow rate, dialysate flow rate, max. blood, min. blood, and stage indicator.

**Table 2:** Comparison of success rates between particle numbers and iteration counts.

Particle #	5 Iter.	15 Iter.	30 Iter.	50 Iter.
1	31%	34%	34%	34%
10	42%	43%	50%	55%
50	65%	69%	70%	72%
100	89%	88%	91%	93%
150	87%	85%	89%	90%
200	91%	88%	91%	93%
300	93%	93%	88%	91%
400	90%	93%	93%	93%

**Table 3:** Feature character comparison before and after applying Particle Swarm Optimization (PSO).

Aspect	Before PSO	After PSO
Value Range	0.00 to ~0.8	0.00 to ~0.8
Color Intensity	Mostly dark purple (low values) with few yellow	More vibrant: magenta, orange, and bright yellow
High-Value Concentration	Sparse, isolated yellow cells	Multiple high-value clusters across filters
Filter Activation Spread	Mostly filters 0–3 show activity	Filters 1–6 show broader activation
Time Step Variation	Time steps 0–2 have most variation	Time steps 1–3 show strong activations

**Table 4:** Comparison statistics between the four federated learning methods with SCAFOLD and FedProx.

Baseline Methods	SCAFFOLD (df = 10)		FedProx (df = 10)	
	t-Value	*p*-Value	t-Value	*p*-Value
Local CNN	0.6746	0.5104	0.7468	0.4671
FedAvg	1.9252	0.0703	1.8494	0.0812
Full PSO (before Server)	9.3783	0.00003	9.3946	0.00002
Full PSO (after Server)	10.9067	0.00001	10.944	0.00003

**Table 5:** Performance comparison of different PSO-based federated learning configurations.

Aggregate Mode	Precision (95%CI)	Recall (95%CI)	F1 Score (95%CI)	Accuracy (%) (95%CI)
Full PSO	0.9143 (0.8253~0.9601)	0.8000 (0.6995~0.8730)	0.8533 (0.7826~0.9068)	90.56 (0.8612~0.9368)
Client PSO	0.8286 (0.7238~0.8991)	0.6591 (0.5553~0.7496)	0.7342 (0.6514~0.8098)	81.97 (0.7653~0.8638)
Server PSO	0.7681 (0.6560~0.8519)	0.5579 (0.4577~0.6536)	0.6463 (0.5595~0.7263)	75.11 (0.6918~0.8022)
Fed-AVG	0.6667 (0.5493~0.7665)	0.4423 (0.3506~0.5381)	0.5318 (0.4417~0.6207)	65.24 (0.5892~0.7106)
SCAFFOLD	0.8034 (0.8311~0.9858)	0.5412 (0.3674~0.5838)	0.6325 (0.5242~0.7314)	79.43 (0.7344~0.8435)
FedProx	0.8112 (0.8232~0.9509)	0.5322 (0.9300~0.9977)	0.6410 (0.8971~0.9740)	80.21 (0.9202~0.9772)
Central model	0.9672 (0.8881~0.9910)	0.7284 (0.6228~0.8133)	0.8310 (0.7519~0.8889)	89.70 (0.8513~0.9298)

**Table 6:** Comparison of related studies (✓: with PSO, ×: without PSO).

Paper	Client-Side PSO	Server-Side PSO	Model Used	Data Set	Performance (Accuracy/AUC etc.)	End Application
[15] **Park et al.**	×	✓	CNN	CIFAR-10, MNIST	70.12% on CIFAR-10	Mobile devices applications
[16] **Li et al.**	✓	×	CNN	FEMNIST, CIFAR-10	81.43% on FEMNIST	Automated Machine Learning
[17] **Ouyang et al.**	×	✓	CNN/VGG16/ResNet	MNIST, FashionMNIST, CIFAR-10, CIFAR-100	~96.8% FedAvg	General comm. Efficiency enhancement
[18] **Elhani et al.**	✓	×	FCAE (Flexible Convolutional Auto-Encoder)	CIFAR-10, MNIST, STL-10, Caltech-101	MNIST 99.51% CIFAR-10 83.5%	General Vision classification
[19] **Kan et al.**	✓	×	1D-CNN (Optimized by APSO)	N-BaIoT Dataset (Mirai & BASHLITE botnets)	Accuracy/F1-Score	IoT Security
[20] **Mirjalili et al.**	✓	×	BPSO (S-shaped/V-shaped)	CEC 2005 Benchmark Functions (25 mathematical functions)	Convergence Speed/Mean Error (Global Optimum)	Global Optimization/Mathematics
[21] **Li et al.**	×	×	CNN/RNN FedProx	FEMNIST Shakespeare, CIFAR-10, synthetic non-IID splits	More robust convergence than FedAvg; improves test accuracy by ~22% in highly heterogeneous settings	mobile/edge devices with system heterogeneity, healthcare, IoT
[22] **Karimireddy et al.**	×	×	CNN/RNN SCAFFOLD	CIFAR-10, EMNIST, Shakespeare, synthetic heterogeneous data	Faster convergence than FedAvg; fewer communication rounds; stable under non-IID data	large-scale federated networks (phones, sensors, hospitals), healthcare
**This paper**	✓	✓	1D-CNN	Hemodialysis Dataset	Global Accuracy: 93%	Hemodialys-is Complication prediction

## Data Availability

The data used in this research remained propriety of the Taichung Veterans General Hospital and remain propriety data of the hospital thus is unavailable due to privacy concern.
